# Lake Metabolism: Comparison of Lake Metabolic Rates Estimated from a Diel CO_2-_ and the Common Diel O_2-_Technique

**DOI:** 10.1371/journal.pone.0168393

**Published:** 2016-12-21

**Authors:** Frank Peeters, Dariia Atamanchuk, Anders Tengberg, Jorge Encinas-Fernández, Hilmar Hofmann

**Affiliations:** 1 Department of Biology, Environmental Physics, University of Konstanz, Konstanz, Germany; 2 Department of Marine Sciences, University of Gothenburg, Gothenburg, Sweden; 3 Department of Oceanography, Dalhousie University, Halifax, Canada; 4 Aanderaa Data Instruments AS, Bergen, Norway; Auckland University of Technology, NEW ZEALAND

## Abstract

Lake metabolism is a key factor for the understanding of turnover of energy and of organic and inorganic matter in lake ecosystems. Long-term time series on metabolic rates are commonly estimated from diel changes in dissolved oxygen. Here we present long-term data on metabolic rates based on diel changes in total dissolved inorganic carbon (DIC) utilizing an open-water diel CO_2_-technique. Metabolic rates estimated with this technique and the traditional diel O_2_-technique agree well in alkaline Lake Illmensee (*pH* of ~8.5), although the diel changes in molar CO_2_ concentrations are much smaller than those of the molar O_2_ concentrations. The open-water diel CO_2_- and diel O_2_-techniques provide independent measures of lake metabolic rates that differ in their sensitivity to transport processes. Hence, the combination of both techniques can help to constrain uncertainties arising from assumptions on vertical fluxes due to gas exchange and turbulent diffusion. This is particularly important for estimates of lake respiration rates because these are much more sensitive to assumptions on gradients in vertical fluxes of O_2_ or DIC than estimates of lake gross primary production. Our data suggest that it can be advantageous to estimate respiration rates assuming negligible gradients in vertical fluxes rather than including gas exchange with the atmosphere but neglecting vertical mixing in the water column. During two months in summer the average lake net production was close to zero suggesting at most slightly autotrophic conditions. However, the lake emitted O_2_ and CO_2_ during the entire time period suggesting that O_2_ and CO_2_ emissions from lakes can be decoupled from the metabolism in the near surface layer.

## Introduction

The balance of the metabolic rates net production, *NEP*, gross primary production, *GPP*, and respiration rate, *R*, is given by:

NEP=GPP–R (1)

Thereby, *R* is defined to assume positive values characterizing respiration. Metabolic rates have not only been defined for individual organisms but also for entire ecosystems or parts of them (e.g., [1–5]).

Lake metabolism describes the turnover of biomass and energy in lake ecosystems. Primary production utilizing light energy to generate chemical energy and converting inorganic carbon into biomass is the basis for the energy flux in food webs and hence is crucial for the understanding of food web dynamics. Respiration, which is associated with oxygen consumption and release of inorganic carbon from the organic carbon pool, may lead to anoxic conditions in the deep-water of lakes and may cause oversaturation of CO_2_ (e.g., [6,7]). The sign of ecosystem net production indicates whether a Lake is a net sink or net source of atmospheric CO_2_. Hence estimates of ecosystem metabolism contribute to the understanding of habitat conditions and food-web dynamics within lake ecosystems as well as of the mass and energy balance of the entire ecosystem. The metabolism of lake ecosystems and of reservoirs is an important factor affecting the carbon flux from terrestrial systems to the ocean and CO_2_ emissions to the atmosphere [8,9]. Estimates of short- and long-term changes in metabolic rates may improve the understanding on how short-term disturbances and long-term environmental change, e.g., climate warming or changes in nutrient loads, may affect the energy and carbon budget of lakes, the fate of terrestrial carbon, and the CO_2_ emission from lakes.

Several techniques have been proposed to measure metabolic rates in aquatic systems (e.g., [10]) and we focus here on open-water techniques utilizing diel changes in dissolved oxygen or carbon [1,11–13]. With the development of oxygen optodes providing reliable long-term data sets on dissolved oxygen at a high temporal resolution (e.g., [14]), the diel O_2_-technique [1] has become widely used to estimate ecosystem metabolism in numerous aquatic systems (e.g., [15] and references in [4,13,16]).

However, the diel O_2_-technique only provides an indirect measure of the metabolic transformations of carbon and the consumption or release of CO_2_. The recent development of CO_2_ optodes [17] opens up the opportunity to utilize long-term data on dissolved CO_2_ concentrations to estimate metabolic rates based on diel changes in dissolved inorganic carbon [18]. Estimates of metabolic rates in lakes utilizing the diel cycle of dissolved inorganic carbon are available for typically only a few days and have been based on diel changes in the concentration of total dissolved inorganic carbon (DIC) measured chemically from collected water samples (e.g., [11,12]) or on diel changes in CO_2_ concentrations neglecting the other components of the carbon balance [2]. The open-water diel CO_2_-technique discussed here enables the estimation of metabolic rates from the diel cycle of DIC concentrations over long time periods at comparatively little field effort. The technique utilizes the combination of a few alkalinity measurements with long-term CO_2_ data measured at sub-hourly resolution to estimate diel changes in DIC concentrations. Such an approach has recently been employed in mesocosm experiments [19] and is adopted here to provide continuous data on carbon based metabolic rates in the surface water of an alkaline lake over several weeks.

Diel CO_2_- and diel O_2_-technique provide independent estimates of lake metabolic rates. However, we hypothesize that the CO_2_-technique is less sensitive to effects by gas exchange than the diel O_2_-technique because the molar atmospheric equilibrium concentration of CO_2_ is much smaller than that of O_2_ and the carbonate balance channels parts of the changes in CO_2_ to carbonate and bi-carbonate.

In the following, we first present the main concepts behind the diel O_2_- and the diel CO_2_-technique and then provide details on the measuring site, instrumentation and deployment of the instruments. After an overview of field data and estimates of metabolic rates covering several weeks at sub-daily resolution, the results are discussed in detail focusing on the comparison of metabolic rates estimated with the diel O_2_- and the diel CO_2_-technique and on the influence of transport processes on these estimates. Supporting information used in this study includes additional data (S1–S3 Appendices), model sensitivity analyses (S4–S7 Appendices), detailed equations (S8 Appendix), and empirical relations (S9 Appendix).

## Methods

### Theory

#### The diel O_2_-technique

The diel O_2_-technique determines net production from the change in the concentration of dissolved oxygen *C*_*O2*_ with time *t*. Assuming that transport and all sources and sinks of dissolved oxygen other than production and respiration can be neglected:

$$\frac{dC_{O2}}{dt}=NEP_{O}(t)=GPP_{O}(t)-R_{O}(t)$$ (2)

The metabolic rates based on the diel O_2_-technique are denoted by subscript O. The effects of transport processes on *C*_*O2*_, e.g., the flux across the air–water interface and vertical mixing, will be discussed later (see Eq 13).

The standard procedure to calculate gross primary production *GPP*_*O*_ from diel changes in dissolved oxygen assumes that the respiration rate *R*_*O*_ is constant during a day [3,20,21] and that *GPP*_*O*_ is zero at night. The night-time respiration rate *R*_*O*_,_*night*_ is commonly estimated from the mean *NEP*_*O*_ during night (e.g., [3]):

$$\begin{array}{ll} R_{O,night} & =-\frac{1}{\Delta t_{night}}\int_{t_{s,night}}^{t_{e,night}}NEP_{O}(t\prime )\cdot dt\prime \\ & \frac{1}{\Delta t_{night}}=\int_{t_{s,night}}^{t_{e,night}}dt\prime =t_{e,night}-t_{s,night}\\ R_{O}(t) & =R_{O,night}\\ GPP_{O}(t) & =NEP_{O}(t)+R_{O,night} \end{array}$$ (3)

Night-time (darkness) and daylight time periods are distinguished on the bases of the timing of dusk, *t*_*dusk*_, and the timing of dawn, *t*_*dawn*_. In the calculations of *R*_*O*.*night*_ the night-time period is commonly defined as the time period between *t*_*s*,*night*_ = *t*_*dusk*_ + Δ*t* and *t*_*e*,*night*_ = *t*_*dawn*_*—Δt* and Δ*t* is here chosen to be one hour to ensure darkness. A day extends from dusk to dusk and the respiration rate *R*_*O*,*night*_ determined for the night starting after the first dusk of this day applies to the entire day until the next dusk.

At night *NEP*_*O*_ and *R*_*O*_,_*night*_ must have opposite sign (Eq 2). Note that the sign convention in Staehr et al. [16, 13] seems to be inconsistent. Note further, that estimates of *R*_*O*,*night*_ based on the mean *NEP*_*O*_ at night utilize the difference between only two O_2_ concentrations in the dissolved oxygen balance, i.e. *C*_*O2*_(*t*_*s*,*night*_) and *C*_*O2*_(*t*_*e*,*night*_):

$$\begin{array}{ll} R_{O,night} & =-\frac{1}{\Delta t_{night}}\int_{t_{s,night}}^{t_{e,night}}NEP_{O}(t\prime )\cdot dt\prime =-\frac{1}{\Delta t_{night}}\int_{t_{s,night}}^{t_{e,night}}\frac{dC_{O2}(t\prime )}{dt\prime }\cdot dt\prime \\ & =-\frac{1}{\Delta t_{night}}\left(C_{O2}\left(t_{e,night}\right)-C_{O2}\left(t_{s,night}\right)\right) \end{array}$$ (4)

The estimate of *R*_*O*,*night*_ based on the mean *NEP*_*O*_ during night may therefore be sensitive to the choice of (*t*_*s*,*night*_) and (*t*_*e*,*night*_) and the errors in the oxygen measurements at these specific times. As an alternative, the estimate of *R*_*O*_,_*night*_ can be based on all dissolved O_2_ measurements during night by using the slope of a linear fit:

$$C_{O2}(t)=a_{O}-R_{O,nightfit}\cdot t\;and\;t_{s,night}<=t<=t_{e,night}$$ (5)

If the original data are collected at a high temporal resolution the derivatives of *C*_*O2*_ are very sensitive to measurement errors and the metabolic rates obtained from such data are rather noisy. Therefore, we smooth the time series of metabolic rates using a simple box-car filter with an averaging period of 6 hours.

#### The diel CO_2_-technique

Metabolic rates based on the diel CO_2_-technique are denoted by subscript C. The diel CO_2_-technique assumes that biomass production is reflected in a loss of carbon from the inorganic carbon pool whereas respiration is associated with an increase in inorganic carbon. Hence, carbon production, *GPP*_*C*_, can be determined from the rate of decrease in the concentration of total dissolved inorganic carbon, *C*_*DIC*_, and the carbon respiration rate *R*_*C*_. The latter can be estimated from the rate of increase in *C*_*DIC*_ at night [11,12]. Making the same assumptions as in the diel O_2_-technique (*GPP*_*C*_,(*t*_*night*_) = 0; *R*_*C*_ = *R*_*C*,*night*_) the metabolic rates based on the balance of inorganic carbon can be determined from:

$$\begin{array}{l} -\frac{dC_{DIC}}{dt}=NEP_{C}(t)=GPP_{C}(t)-R_{C}(t)\\ R_{C,night}=-\frac{1}{\Delta t_{night}}\int_{t_{s,night}}^{t_{e,night}}NEP_{C}(t\prime )\cdot dt\prime \\ R_{C}(t)=R_{C,night}\\ GPP_{C}(t)=NEP_{C}(t)+R_{C,night} \end{array}$$ (6)

As in the diel O_2_-technique night-time respiration rate *R*_*C*,*night*_ can be determined from the mean *NEP*_*C*_ at night or from linear regression:

$$R_{C,night}=\frac{1}{\Delta t_{night}}\left(C_{DIC}\left(t_{e,night}\right)-C_{DIC}\left(t_{s,night}\right)\right)$$ (7)

$$C_{DIC}(t)=a_{C}+R_{C,nightfit}\cdot t\;and\;t_{s,night}<=t<=t_{e,night}$$ (8)

These equations for the assessment of metabolic rates from diel changes in *C*_*DIC*_ are essentially the same as for the diel O_2_-technique, but the net production is based on the rate of change of *DIC* rather than that of O_2_, and the relations between the rate of concentration change and the metabolic rates have opposite sign compared to the diel O_2_-technique.

The calculation of the metabolic rates with the diel CO_2_-technique requires data on *C*_*DIC*_ at sub-daily resolution. Because *C*_*DIC*_ can be estimated from concentrations of CO_2_ if *pH* is known (see further below), CO_2_ measurements with high temporal resolution available from CO_2_-optodes can be utilized to estimate metabolic rates. This is the basis of the diel CO_2_-technique.

#### Estimation of time series of *C*_*DIC*_ from *C*_*CO2*_ data

CO_2_-sensors typically provide the partial pressure of CO_2_, i.e. *pCO*_*2*_. The sum of the concentrations of dissolved CO_2(aq)_ and un-dissociated hydrated CO_2_(H_2_CO_3(aq)_) in the sampled water, *C*_*CO2*_ in this study, can be determined from *pCO*_*2*_ using Henry’s Law. The Henry coefficient, *H*, depends on the water temperature *T* and salinity *S* and was calculated from the empirical relations of Weiss [22]. The calculation of *C*_*DIC*_ from *C*_*CO2*_ is straight forward if the *pH* of the water is known [23]:

$$C_{CO2}=H\left(T,S\right)\cdot pCO_{2}$$ (9)

$$C_{DIC}=C_{CO2}/\alpha_{0}\left(pH,T,S\right)$$ (10)

The coefficient *α*_*0*_ depends on *pH*, *T*, and *S* (see Table in S9 appendix). Values of *pH* typically show daily cycles in response to production and respiration. The values of *pH* also change if CO_2_ is introduced or removed by gas exchange, e.g., with the atmosphere. Hence, the calculation of *C*_*DIC*_ from *C*_*CO2*_ and *pH* requires precise data on *pH* at sub-daily resolution over long time periods. Unfortunately, submersible in-situ *pH*-sensors that can be deployed for several weeks and have sufficient long-term stability, absolute accuracy and precision are currently difficult to encounter. Therefore, it is advantageous to base the calculation of *C*_*DIC*_ from *C*_*CO2*_ data on measurements of alkalinity rather than on *pH* measurements (see also [24]). The *pH* values required for the calculation of *C*_*DIC*_ can be estimated from carbonate alkalinity *ALK*_*Carb*_ [mmol_eq_ L^-1^] and *C*_*CO2*_ [mmol L^-1^]:

$$ALK_{Carb}=C_{HCO3-}+2\cdot C_{CO3--}+C_{OH-}–C_{H3O+}$$ (11)

$$C_{DIC}=C_{CO2}/\alpha_{0};C_{HCO3-}=C_{DIC}\cdot \alpha_{1};C_{CO3--}=C_{DIC}\cdot \alpha_{2}$$

$$ALK_{Carb}=C_{CO2}/\alpha_{0}(\alpha_{1}+2\cdot \alpha_{2})+\left(K_{W}\cdot 10^{pH}–10^{-pH}\right)\cdot 1000$$ (12)

whereby *C*_*HCO3-*_ and *C*_*CO3—*_ are the concentrations of HCO_3_^-^ and CO_3_^--^, respectively, *C*_*OH-*_ and *C*_*H3O+*_ the concentrations of OH^-^ and H_3_O^+^ ions. The coefficients *α*_*1*_and *α*_*2*_ depend on *pH*, *T*, and *S*. The empirical relations for *α*_*1*_, *α*_*2*_ and *K*_*W*_ are listed in Table in S9 appendix. Eq (12) is an implicit equation for *pH*.

Alkalinity may change in case of calcite precipitation and dissolution of solid carbonates but also due to several other biogeochemical processes ([25]). However, changes in *C*_*CO2*_ due to gas exchange with the atmosphere or due to uptake or release by phytoplankton during production and respiration, respectively, do not alter alkalinity [25] because the dissociation of H_2_CO_3(aq)_ to negatively charged carbonate ions is associated with the generation of an equivalent number of positively charged hydronium ions. Also nutrient uptake by phytoplankton has only minor effects on alkalinity [19]. Phosphate and nitrate assimilated during primary production or released during remineralization of organic material alter alkalinity [25] but the molar fraction of phosphate and nitrogen in phytoplankton is rather small (i.e. the typical ratios C:N:P = 106:16:1 [26]). Hence, if the only processes affecting inorganic carbon are production/respiration and gas exchange of CO_2_ with the atmosphere, the carbonate alkalinity *ALK*_*carb*_ can be treated as essentially conserved quantity. Then, *pH* and the daily cycle of *pH* can be calculated from a single measurement of *ALK*_*carb*_ and the time series of *pCO*_*2*_.

All coefficients in Eq (12) depend on *T* and *S*, and *α*_*0*_, *α*_*1*,_ *α*_*2*_ additionally on *pH*. If *T*, *S*, *C*_*CO2*_ and *ALK*_*carb*_ are known, *pH* can be calculated from Eq (12) by solving this implicit equation numerically. We employ a least squares fitting procedure varying *pH* to minimize the root mean square difference between calculated and measured *ALK*_*carb*_ (fminsearch of MATLAB using the Nelder Mead simplex algorithm). With the *pH* determined from Eq (12), *α*_*0*_ can be calculated and *C*_*DIC*_ be determined from Eq (10).

#### Considering vertical transport

In lake ecosystems, temporal changes in the concentrations of dissolved O_2_ and DIC are caused not only by metabolic processes but also by transport processes. Assuming horizontally homogeneous conditions, the temporal change of the vertical distribution of *C*_*O2*_ considering metabolic processes and vertical fluxes due to transport processes is given by:

$$\begin{array}{l} \frac{\partial C_{O2}}{\partial t}=GPP_{O}-R_{O}-\frac{1}{A}\frac{\partial \left(A\cdot F_{O2}\right)}{\partial z}+\frac{1}{A}\frac{\partial A}{\partial z}F_{O2,sed}\\ Boundary\;conditions:\\ F_{O2,surf}=v_{O2}\cdot \left(C_{O2}-C_{O2,equ}\right)\\ F_{O2,bot}=F_{O2,sed} \end{array}$$ (13)

where *C*_*O2*_ is the concentration of dissolved oxygen as function of *z*, *z* is the vertical coordinate (positive in the upward direction), *A* is the cross-section at *z*, *F*_*O2*_ is the vertical flux of dissolved oxygen at *z*, *F*_*O2*,*sed*_ is the flux of O_2_ from the sediments at *z* into the water, *F*_*O2*,*surf*_ and *F*_*O2*,*bot*_ are the fluxes of O_2_ in direction *z* at the surface and at the bottom boundary, respectively. At the bottom *F*_*O2*,*bot*_ = *F*_*O2*,*sed*_. At the surface, *F*_*O2*,*surf*_ is determined by the flux due to gas exchange with the atmosphere *F*_*O2*,*atm*_. *C*_*O2*,*equ*_ is the equilibrium concentration of O_2_ at ambient surface water temperature and salinity and atmospheric pressure, *v*_*O2*_ is the gas exchange velocity of dissolved oxygen.

Within the sediments dissolved O_2_ is consumed by bacteria that mineralize organic material which typically results in anoxic conditions in deeper lake sediments. Hence, *F*_*O2*,*sed*_ is typically negative and acts as a sink of dissolved O_2_ in the water column. In lake metabolism studies this sedimentary flux is often not explicitly considered (e.g., [3,5]) and thus implicitly included in the system respiration rate. The commonly used lake respiration rate *R*_*L_O*_ therefore is:

$$R_{L\_O}=R_{O}-\frac{1}{A}\frac{\partial A}{\partial z}F_{O2,sed}$$ (14)

Additionally, the oxygen loss due to the flux at the lake bottom is also attributed to the system respiration rate and included in *R*_*L_O*_ by assuming a zero-flux boundary condition at the lake bottom (*F*_*O2*,*bot*_ = 0). The equation for *NEP*_*L_O*_ becomes:

$$NEP_{L\_O}=\frac{\partial C_{O2}}{\partial t}+\frac{1}{A}\frac{\partial \left(A\cdot F_{O2}\right)}{\partial z}$$ (15)

with *F*_*O2*,*surf*_ = *F*_*O2*,*atm*_ and *F*_*O2*,*bot*_ = 0 as boundary conditions.

The budget of dissolved inorganic carbon can be described analogously:

$$\begin{array}{l} \frac{\partial C_{DIC}}{\partial t}=-GPP_{C}+R_{C}-\frac{1}{A}\frac{\partial \left(A\cdot F_{DIC}\right)}{\partial z}+\frac{1}{A}\frac{\partial A}{\partial z}F_{DIC,sed}\\ Boundary\;conditions:\\ F_{DIC,surf}=F_{CO2,surf}=v_{CO2}\cdot \left(C_{CO2}-C_{CO2,equ}\right)\\ F_{DIC,bot}=F_{DIC,sed} \end{array}$$ (16)

where *F*_*DIC*_ is the vertical flux of inorganic carbon, *F*_*DIC*,*sed*_ is the flux of DIC from the sediments into the water column, *F*_*DIC*,*surf*_ and *F*_*DIC*,*bot*_ are the fluxes of DIC in direction *z* at the surface and the bottom boundary, respectively. The fluxes, concentrations and metabolic rates are functions of z.

At the bottom, *F*_*DIC*,*bot*_ = *F*_*DIC*,*sed*_. At the surface, the flux of DIC is the flux of CO_2_ due to gas exchange with the atmosphere, *F*_*CO2*_. *C*_*CO2*,*equ*_ is the equilibrium concentration of CO_2_ at ambient water temperature and salinity and atmospheric pressure, *v*_*CO2*_ is the gas exchange velocity of CO_2_.

Note that gross primary production is a source of dissolved oxygen whereas it is a sink of DIC, which is accounted for by the opposite signs in Eqs (13) and (16). Note further, that in case of DIC the surface flux is determined by *C*_*CO2*_ only and not by *C*_*DIC*_.

In analogy to the system metabolic rates based on dissolved oxygen one can define system metabolic rates based on carbon that include mineralization of organic material in the sediments and sediment fluxes into the system respiration rate:

$$R_{L,C}=R_{C}+\frac{1}{A}\frac{\partial A}{\partial z}F_{DIC,sed}$$ (17)

$$NEP_{L\_C}=-\frac{\partial C_{DIC}}{\partial t}-\frac{1}{A}\frac{\partial \left(A\cdot F_{DIC}\right)}{\partial z}$$ (18)

with *F*_*DIC*,*surf*_ = *F*_*CO2*,*atm*_ and *F*_*DIC*,*bot*_ = 0 as boundary conditions. Note the opposite sign in Eq (18) compared to Eq (15).

In the following we determine *NEP*_*L_O*_ and *NEP*_*L_C*_ from Eqs (15) and (18), respectively, and test the consequences of several assumptions regarding the vertical fluxes of dissolved oxygen and of DIC:

1. As the simplest approach we assume that the gradients of the vertical fluxes are zero, i.e. that the vertical fluxes due to transport processes in the water column are independent of depth and agree with the flux at the lake surface.
  $$\frac{1}{A}\frac{\partial \left(A\cdot F_{O2}\right)}{\partial z}=0$$ (19)
2. The second approach includes gas exchange with the atmosphere at the lake surface but neglects all other transport. This approach was used by, e.g., Cole et al. [20] and was recommended by Staehr et al. [16] for experiments in which measurements are available only from one water depth. The change in concentration due to the gas exchange at the lake surface can be estimated assuming a mixed surface layer with depth *Z*_*mix*_ [16,20,21]. *Z*_*mix*_ is estimated from temperature profiles as outlined in S1 appendix. The volume of the mixed surface layer is *V*_*m*ix_ and the surface area *A*_*o*_.
  $$\frac{1}{A}\frac{\partial \left(A\cdot F_{O2}\right)}{\partial z}=\frac{F_{O2,atm}\cdot A_{0}}{V_{mix}}$$ (20)
3. The third approach considers the full mass balance of O_2_ in the surface mixed layer by including not only the fluxes of O_2_ at the lake surface due to gas exchange with the atmosphere but also the fluxes at the bottom boundary of the mixed surface layer, i.e. at *Z*_*mix*_, (*F*_*O2*,*Zmix*_) due to mixing processes. The flux *F*_*O2*,*Zmix*_ is assumed to comprise of fluxes due to turbulent diffusion, *F*_*O2*,*turb*_, and fluxes associated with mixed layer deepening, *F*_*O2*,*deepen*_:
  $$\begin{array}{l} F_{O2,turb}=-K_{z}\frac{dC_{O2}}{dz}\\ F_{O2,deepen}=\frac{1}{\Delta t}\frac{1}{A_{Zmix}}\left(\frac{1}{V_{Zmix(2)}}\int_{-Zmix(2)}^{0}A\cdot C_{O2}\cdot dz\prime -\frac{1}{V_{Zmix(1)}}\int_{-Zmix(1)}^{0}A\cdot C_{O2}\cdot dz\prime \right) \end{array}$$ (21)
  $$F_{O2,Zmix}=\{\begin{array}{cc} F_{O2,turb} & ifdZ_{mix}/dt<=0\\ F_{O2,turb}+F_{O2,deepen} & ifdZ_{mix}/dt>0 \end{array}$$
  $$\frac{1}{A}\frac{\partial \left(A\cdot F_{O2}\right)}{\partial z}=\frac{A_{0}\cdot F_{O2,atm}-A_{Zmix}\cdot F_{O2,Zmix}}{V_{Zmix}}$$ (22)

Turbulent diffusion coefficients *K*_*z*_ were calculated as in Staehr et al. [3] from the empirical relation of Hondzo and Stefan [27] using data from a thermistor chain (see S1 and S2 Appendices). Vertical gradients of *C*_*O2*_ at *Z*_*mix*_ were determined by linear interpolation of the gradients of *C*_*O2*_ obtained from O_2_-measurements at 1.2 m, 3.2 m and 5.2 m depth. *A*_*Zmix*_, is the area of the cross section at *Z*_*mix*_. The oxygen profile at time 1, *C*_*O2*_, was integrated from *Z*_*mix*_ at time 1, *Z*_*mix*_ (1), to the surface and from *Z*_*mix*_ after the time interval Δ*t*, i.e. from *Z*_*mix*_ (2) at time 2, to the surface. The time interval Δ*t* was chosen to be one hour which allows resolving day-night changes in *Z*_*mix*_ while avoiding influences from measurement noise and high-frequency oscillations.

The lake net production rates for the different approaches are:

$$NEP_{L\_O}=+\frac{\partial C_{O2}}{\partial t};\;NEP_{L\_C}=-\frac{\partial C_{DIC}}{\partial t}$$ (23i)

$$NEP_{L\_O,A}=+\frac{\partial C_{O2}}{\partial t}+\frac{F_{O2,atm}\cdot A_{0}}{V_{mix}};\;NEP_{L\_C,A}=-\frac{\partial C_{DIC}}{\partial t}-\frac{F_{CO2,atm}\cdot A_{0}}{V_{mix}}$$ (23ii)

$$NEP_{L\_O,\text{F}}=+\frac{\partial C_{O2}}{\partial t}+\frac{A_{0}\cdot F_{O2,atm}-A_{Zmix}\cdot F_{O2,Zmix}}{V_{Zmix}}$$ (23iii)

$$NEP_{L\_O,D}=+\frac{\partial C_{O2}}{\partial t}+\frac{A_{0}\cdot F_{O2,atm}-A_{Zmix}\cdot F_{O2,turb}}{V_{Zmix}}$$ (23iv)

The metabolic rates determined with the approaches (ii) and (iii) are indicated by subscript labels *A* and *F*, respectively. Metabolic rates estimated from approach (iv) that adopts approach (iii) but neglects fluxes due to mixed layer deepening are labeled with subscript *D*. Eq (23)) requires estimates of *C*_*O2*,*equ*_, *C*_*CO2*,*equ*_, *v*_*O2*_, and *v*_*CO2*_. The equilibrium concentrations were determined from [28] in case of O_2_ and from [22] in case of CO_2_. Gas exchange velocities were calculated by combining the empirical relation of Cole and Caraco [29] for the gas-exchange velocity of CO_2_ in freshwater at 20°C (i.e. at Schmidt number *S*_*C*_ = 600) with the Schmidt number dependence of the gas-exchange velocity suggested by Liss and Merlivat [30]. The Schmidt number dependence is required to include the effect of temperature on the gas-exchange velocity and also allows using the same parametrization of the gas-exchange velocity for CO_2_ and O_2_.

From the *NEP*_*L_O*_ and *NEP*_*L_C*_ the other metabolic rates (*R*_*L_O*_, *GPP*_*L_O*_, *R*_*L_C*_, *GPP*_*L_C*_) were calculated assuming that during each day the lake respiration rate remains constant and that lake gross primary production is zero at night. Hence, the lake respiration rate is equal to the negative of the lake net production during the night of the respective day (*R*_*L_C*_ = -*NEP*_*L_C*,*night*_ and *R*_*L_O*_ = -*NEP*_*L_O*,*night*_). The respiration rates can be obtained by averaging:

$$\begin{array}{l} R_{L\_O,night}=-\frac{1}{\Delta t_{night}}\int_{t_{s,night}}^{t_{e,night}}NEP_{L\_O}(t\prime )\cdot dt\prime \\ R_{L\_C,night}=-\frac{1}{\Delta t_{night}}\int_{t_{s,night}}^{t_{e,night}}NEP_{L\_C}(t\prime )\cdot dt\prime \end{array}$$ (24)

or by the application of linear regression to flux modified concentrations *C*_*O2*,*mod*_ and *C*_*DIC*,*mod*_:

$$\begin{array}{l} C_{O2,\text{mod}}(t)=C_{O2}(t)+\int_{t_{s,night}}^{t}\frac{1}{A}\frac{\partial \left(A\cdot F_{O2}(t\prime \right)}{\partial z}\cdot dt\prime \\ C_{O2,\text{mod}}(t)=a_{L\_O}-R_{L\_O,nightfit}\cdot t\;and\;t_{s,night}<=t<=t_{e,night}\\ C_{DIC,\text{mod}}(t)=C_{DIC}(t)+\int_{t_{s,night}}^{t}\frac{1}{A}\frac{\partial \left(A\cdot F_{DIC}(t\prime \right)}{\partial z}\cdot dt\prime \\ C_{DIC,\text{mod}}(t)=a_{L\_C}+R_{L\_C,nightfit}\cdot t\;and\;t_{s,night}<=t<=t_{e,night} \end{array}$$ (25)

Daily mean metabolic rates were calculated for days at which at least 23 hours of data were available (55 days for the diel O_2_- and 50 days for the diel CO_2_- technique). Long-term averages of metabolic rates were calculated from daily mean metabolic rates considering only 49 days for which data were available from the diel O_2_- and the diel CO_2_-technique.

### Field experiments

In 2014 field experiments were conducted in Lake Illmensee, a small (surface area: 64 ha, maximum water depth: 16.5 m) alkaline (*pH* of ~8.5) lake located in southern Germany (47° 51’ 19” N, 9° 22’ 49”E) at 670 m above sea level. The field studies did not involve endangered or protected species and were permitted by the Landratsamt Sigmaringen. From May 26^th^ to July 28^th^ moorings were installed at the deepest station of the lake. The moorings were equipped with thermistors (RBRsolo T, RBR) measuring temperature every 10 s and eight O_2_-optodes (MiniDOT, PME, accuracy ~-10 μmol L^-1^) measuring every 60 s dissolved oxygen concentrations (*C*_*O2*_). The O_2_ data were calibrated by scaling O_2_ measurements in air to provide 100% saturation. One of the temperature loggers additionally had a pressure sensor (TDR, RBR) that was used to measure the height of the water column above the sensor and air pressure during lifts of the mooring. The vertical spacing of the O_2_-optodes was 2 m and of the thermistors 1 m. The uppermost O_2_-optode and thermistor were mounted at ~1.2 m water depth. At ~1.7 m water depth a CO_2_-optode (Aanderaa Data Instruments, Norway; Atamanchuk et al. [17]) measured *pCO*_*2*_ and temperature every 30 s during the entire time period. The data from the CO_2_-optode was stored in a data logger built by the electronic workshop at the University of Konstanz. Another CO_2_-sensor based on IR absorption spectroscopy (HydrocC^™^ CO_2_, Contros; in the following: CO_2_-IRprobe) was mounted at 2 m water depth and measured *pCO*_*2*_ every 5 s. The CO_2_-IRprobe had comparatively large power consumption and was therefore deployed for continuous measurements only from June 23^rd^ 4 pm to June 28^th^ 12 am requiring one battery change during this 4.8 day time period. The CO_2_-optode required only one battery change during the 63 days of deployment. Breaks in the time series of *pCO*_*2*_ data from the CO_2_-optode resulted from lifting the mooring for maintenance of the other instruments. The pCO_2_ data from the pre-calibrated CO_2_-optode were corrected for the conditioning effect by introducing a single constant scaling factor [17]. The calibration of this scaling factor was based on the data from the CO_2_-IRprobe. The conditioning effect results from chemical reactions between the foil of the CO_2_-optode and the ambient water when the foil is deployed for the first time [17].

On June 23^rd^ and June 30^th^ a vertical profile of water samples was collected at the deepest station. Total alkalinity was measured by titration. *ALK*_*carb*_ was assumed to correspond to the total alkalinity. On 23^rd^ June and July 1^st^ vertical profiles of *pCO*_*2*_ including atmospheric partial pressures of CO_2_ were measured with the CO_2_-IRprobe. At each depth the CO_2_-IRprobe was deployed for 20 minutes allowing adjustment of the probe to the high concentrations at larger water depths. Wind speed was measured every 15 minutes 1.5 m above the lake water level on a buoy installed close to the deepest station of the lake (ISF Langenargen). Wind speed at 10 m above lake level *WS*_*10*_ was calculated from these wind speed data assuming a log-boundary layer, wind speed dependent drag coefficients *C*_*10*_ according to Wu [31] and assuming *C*_*10*_ ≥ 10^−3^ (S1 appendix). Further, profiles were taken with a multi-parameter CTD (RBR) equipped with an oxygen optode (fast optode model 4330F, Aanderaa Data Instruments, Norway), Chl.-*a* sensor (Seapoint), two PAR sensors (Licor) and a turbidity sensor (Seapoint), and with a multi-spectral fluorescence probe (Moldaenke FluoroProbe).

## Results

**Fig 1:**
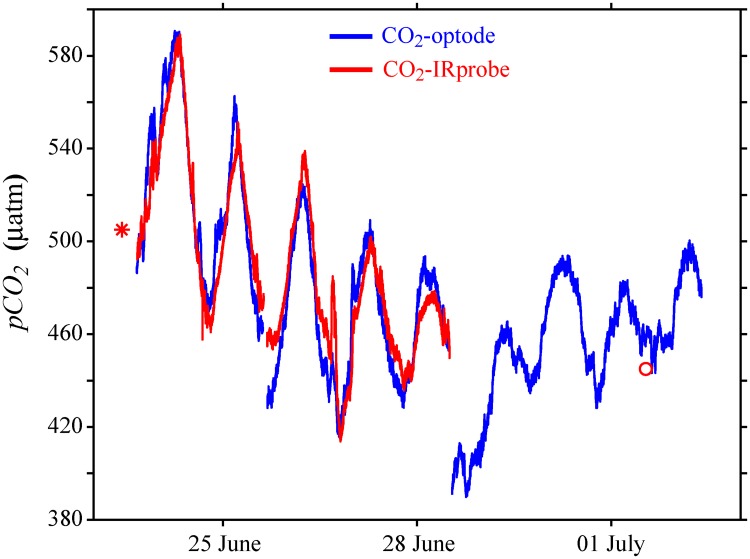
Comparison of time series on *pCO*_*2*_ measured with the CO_2_-optode (blue line) at 1.7 m water depth and the CO_2_-IRprobe (red line) at 2.0 m water depth. The red symbols represent additional individual measurements with the CO_2_-IRprobe.

The values of *pCO*_*2*_ in air measured with the CO_2_-IRprobe on 23^rd^ June and 1^st^ July were 364 and 352 μatm, respectively. These values correspond to 394 and 382 ppm at local air pressure of 0.924 and 0.922 atm, respectively, and thus agree well with the current atmospheric concentration of ~400 ppm CO_2_ [32]. The long-term changes and the amplitude of the daily fluctuations of *pCO*_*2*_ measured with the CO_2_-optode agree well with those measured with the CO_2_-IRprobe (Fig 1). The good agreement of the amplitude and the timing of the daily fluctuations in *pCO*_*2*_ measured with the CO_2_-optode and the CO_2_-IRprobe support that the CO_2_-optode provides reliable data on *pCO*_*2*_ over an extended period of time. Four days after the calibration period the CO_2_-optode still agreed well with an independent measurement of the CO_2_-IRprobe (Fig 1, red circle).

**Fig 2:**
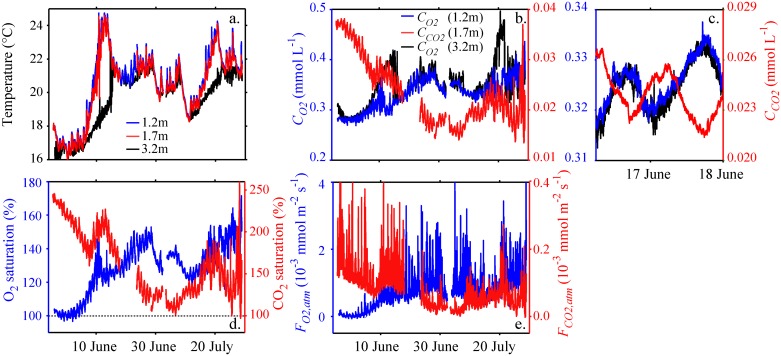
Surface water temperature and concentrations, saturation, and surface fluxes of O_2_ and CO_2_. Temperature (a) and concentrations of dissolved O_2_ and dissolved CO_2_ (b and c) were measured with the O_2_-optodes at 1.2 m (blue) and 3.2 m (black) water depth and the CO_2_-optode at 1.7 m water depth (red). (c) depicts an enlargement of (b) to illustrate details of the daily changes in *C*_*O2*_ and C_CO2_. Both, O_2_ and CO_2_ concentrations are oversaturated compared to atmospheric equilibrium at in-situ temperature during most of the time (d). The flux of O_2_ (*F*_*O2*,*atm*_) and CO_2_ (*F*_*CO2*,*atm*_) to the atmosphere is depicted in panel (e). O_2_-saturation and *F*_*O2*,*atm*_ (blue lines in (d) and (e)) are based on the *C*_*O2*_ data measured at 1.2 m water depth. The large short-term fluctuations in the fluxes to the atmosphere result from the variation in wind speed (see Fig Panel a in S1 appendix).

Water temperatures increased at the beginning of the measuring period and were around 22°C thereafter (Fig 2a). The water temperatures at the water depths of the uppermost O_2_-optode (1.2 m) and of the CO_2_-optode (1.7 m) were essentially the same (blue and red lines in Fig 2a) indicating that the top 1.7 m of the water column was rather homogeneously mixed. This conclusion is consistent with the typical values for the mixed layer depth *Z*_*mix*_ (average *Z*_*mix*_ is 2.9 m, Fig Panel c in S1 appendix). The water temperatures measured with the O_2_-optode located at 3.2 m water depth (Fig 2a, black line) were similar to the temperatures at 1.2 and 1.7 m depth but were substantially lower between the 7^th^ and 15^th^ of June and between the 16^th^ and 21^st^ of July. During these time periods *Z*_*mix*_ was smaller than 3.2 m (Fig Panel c in S1 appendix).

The temporal development of *C*_*O2*_ and of *C*_*CO2*_ was typically anti-correlated at time scales of several days but also at sub-daily time scales (Fig 2b and 2c). Both, *C*_*O2*_ and *C*_*CO2*_, showed daily concentration fluctuations consistent with metabolic transformations during different time periods of the day: *C*_*O2*_ was elevated during daytime and reduced during night-time whereas *C*_*CO2*_ showed the opposite pattern (Fig 2b and 2c). *C*_*O2*_ measured at 1.2 m and at 3.2 m water depth agreed well when temperatures agreed well and *Z*_*mix*_ was larger than 3.2 m, but during time periods with *Z*_*mix*_ < 3.2 m *C*_*O2*_ at 3.2 m depth was larger than at 1.2 m depth (Fig 2a and 2b, blue and black lines and Fig Panel c in S1 appendix). Below 3.2 m water depth O_2_ concentrations increased substantially with depth during most of the time period reaching maximum values at ~7 m depth (Fig Panels b and c in S2 appendix and Fig Panel f in S3 appendix). Below the peak concentration O_2_ decreased rapidly to anoxic conditions in the deep water. The vertical O_2_-gradients were small initially but they increased substantially between the 7^th^ and 10^th^ of June, when very high O_2_ concentrations developed at intermediate depths (Fig Panel b in S2 appendix).

During the measuring period CO_2_ and O_2_ near the lake surface were typically oversaturated (Fig 2d). Hence, the lake emitted carbon and oxygen to the atmosphere. During most of the measuring period, the daily fluctuations in the oversaturation of CO_2_ and O_2_ were small compared to the total oversaturation suggesting that the emissions were not controlled by the daily metabolic cycle during the time period of measurements (Fig 2d). Note that the molar fluxes of O_2_ to the atmosphere were substantially larger than those of CO_2_ (Fig 2e), although the oversaturation of CO_2_ was much larger than that of O_2_ (Fig 2d). On average the emissions of O_2_ and CO_2_ were 64 mmol m^-2^ d^-1^ and 7 mmol m^-2^ d^-1^, respectively. During the measuring period no extreme wind events occurred and wind speeds were typically below 10 m s^-1^ (Fig Panel a in S1 appendix). The O_2_ oversaturation in the surface water increased substantially at the beginning of June. The timing of this change in oversaturation corresponds closely with the onset of the development of the dissolved oxygen peak at ~7 to 8 m water depth (Fig 2d and Fig Panel b in S2 appendix and Fig Panel f in S3 appendix). Note that the O_2_-optodes were located at 7.2 and 9.2 m water depth and that the maximum O_2_ concentration measured with the O_2_ sensor of the CTD-probe was at ~8 m depth.

Profiles of Chl_a_-equivalent concentration measured with the multi-spectral fluorescence probe showed a pronounced maximum at ~8 m depth (Fig Panel g in S3 appendix). Analysis of water samples and the spectral information from the fluorescent probe suggest that this peak in the Chl_a_-equivalent concentration was generated by a dense layer of *Plankthotrix rubescens* (see [33] for measuring *P*. *rubescens* with the Moldaenke FluoroProbe).

**Fig 3:**
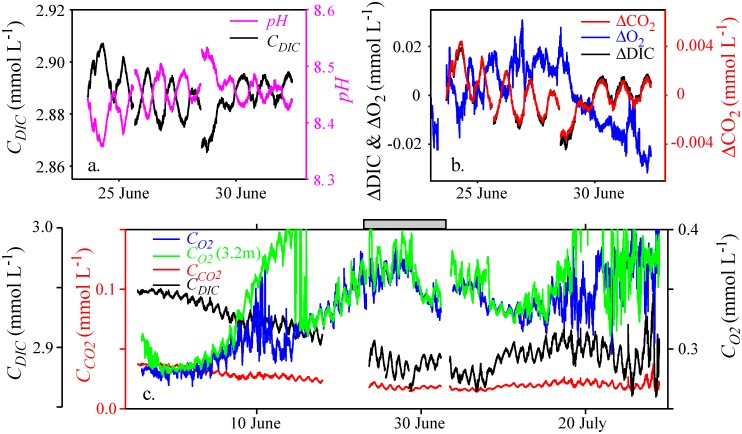
Comparison of the temporal development of DIC, *pH*, CO_2_ and O_2_ concentrations. (a) *C*_*DIC*_ and *pH* derived from *C*_*CO2*_ and a constant alkalinity of 2.95 mmol_eq_ L^-1^. (b) Deviation of DIC, O_2_ and CO_2_ concentrations from the respective mean concentration within the time interval shown (ΔDIC, ΔO_2_ and ΔCO_2_, respectively). Note that the scaling of the axis for the molar deviations ΔDIC and ΔO_2_ is five times larger than the scaling of the axis for ΔCO_2_. (c) Long-term changes of *C*_*DIC*_, *C*_*CO2*_ and *C*_*O2*_. In (c) y-axes have shifted origin but the same scaling. The grey bar in (c) indicates the time period depicted in (a) and (b).

At 2 m water depth alkalinity was 2.98 mmol_eq_ L^-1^ on June 23^rd^ and 2.93 mmol_eq_ L^-1^ on June 30^th^, suggesting that alkalinity did not change substantially over this one-week time period. In the following we use 2.95 mmol_eq_ L^-1^ as value for *Alk*_*Carb*_ during the entire measuring period. The time series of *pH* calculated from *Alk*_*Carb*_, *pCO*_*2*_ and *T* shows periodic fluctuations. Within a day the values of *pH* varied by ~0.1 (Fig 3a). For the time period shown in Fig 3a the average *pH* was ~8.45. *C*_*DIC*_ determined from the estimated time series of *pH* and the measured time series of *pCO*_*2*_ and *T* typically decreases during the day and increases at night (Fig 3a). The daily changes in DIC and O_2_ concentrations are anti-correlated, i.e. *C*_*O2*_ increases while *C*_*DIC*_ decreases during daylight time and vice versa during night-time (Fig 3b). The amplitudes of the daily fluctuations in *C*_*DIC*_ are about the same as those in *C*_*O*2_ at 1.2 m and 3.2 m water depth but are about 5 times larger than the amplitudes of the daily fluctuations in *C*_*CO2*_. This indicates that a substantial fraction of the dissolved inorganic carbon taken up and released during production and respiration alters HCO_3_^-^ and CO_3_^--^ concentrations much more than CO_2_ concentrations. However, the amplitude of the *C*_*DIC*_ fluctuations is less than 1% of the daily mean *C*_*DIC*_. Neglecting the daily fluctuations of *pH* in the calculation of *C*_*DIC*_ leads to ~20 times larger amplitudes of the daily fluctuations of *C*_*DIC*_ (Fig in S4 appendix) and thus would result in a severe overestimation of *NEP*_*L*_*_*_*C*_.

**Fig 4:**
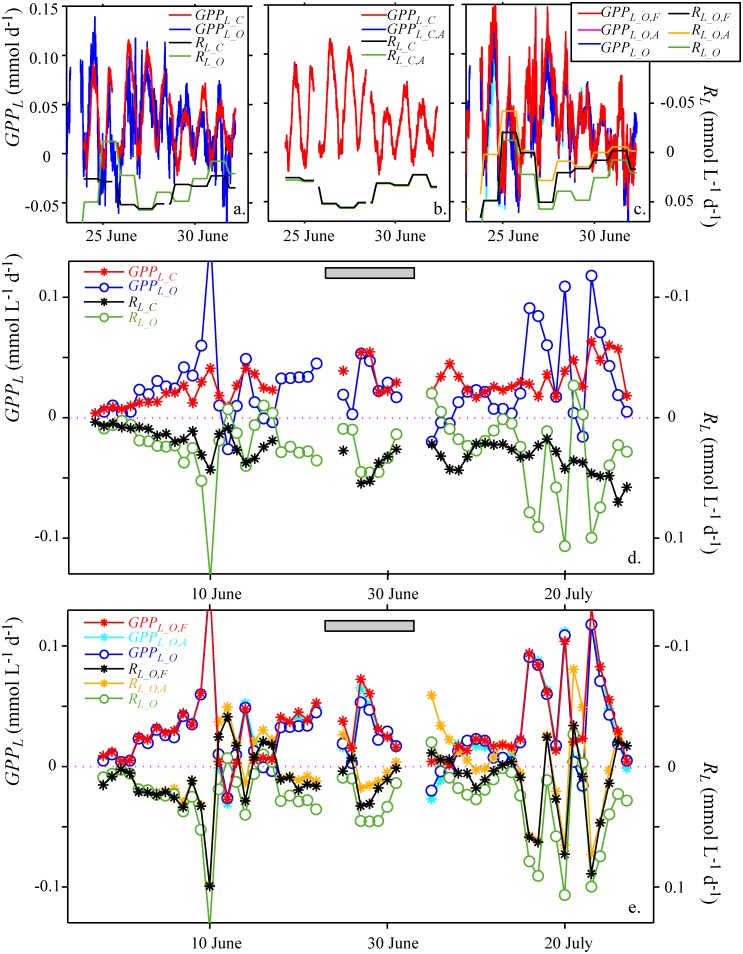
Comparison of lake metabolic rates estimated with the diel CO_2_- and the diel O_2_-technique. (a) Comparison of box-car filtered lake gross primary production *GPP*_*L*_ and lake respiration rate *R*_*L*_ estimated with both techniques. (b) Comparison of the effect of different assumptions on vertical transport on *GPP*_*L*_ and *R*_*L*_ (approaches (i)-(iii) and Eqs 23i–23iii in the methods section) estimated with the diel CO_2_-technique (*GPP*_*L_C*_ and *R*_*L_C*_). (c) as in (b) but for *GPP*_*L*_ and *R*_*L*_ estimated with the diel O_2_-technique (*GPP*_*L_O*_ and *R*_*L_O*_). (d) Long-term changes in daily mean lake metabolic rates estimated with both techniques assuming that the net fluxes are zero (approach (i)). (e) Implications of different assumptions on the vertical fluxes for the daily mean metabolic rates estimated with the diel O_2_-technique. *GPP*_*L_O*,*A*_ is often covered by *GPP*_*L_O*_ and *GPP*_*L_O*,*F*._ Note that in all panels lake respiration rates are represented using a reverse axis, i.e. *R*_*L*_ is increasing in the downward direction. The grey bar indicates the time period shown in panels a-c.

Lake metabolic rates determined from O_2_ and CO_2_ measurements are shown in Fig 4. Lake respiration rates were determined from linear regression of lake net production as function of time during night-time (Eq (25)). These respiration rates agree well with respiration rates estimated by averaging lake net production during night-time as in Eq (24) (Fig Panel a in S5 appendix).

Lake gross primary production (*GPP*_*L*_) shows a pronounced daily cycle with minimum values occurring around midnight and maximum values around noon (Fig 4a). The phase and amplitude of the daily cycles of *GPP*_*L_O*_ and *GPP*_*L_C*_ are similar (Fig 4a). In the diel O_2_- and diel CO_2_-techniques lake respiration rates (*R*_*L*_) are assumed to be constant during a day. The order of magnitude and the temporal changes in *R*_*L_O*_ and *R*_*L_C*_ are similar, but *R*_*L_O*_ shows larger fluctuations between days than *R*_*L_C*_ (Fig 4a and 4d), especially around the 10^th^ of June and the 20^th^ of July. The long-term average and the long-term trends of daily mean *GPP*_*L_O*_ and *GPP*_*L_C*_ agree well (Table 1, Fig 4d), but the daily mean *GPP*_*L_O*_ fluctuate more between days than the daily mean *GPP*_*L_C*_. *R*_*L_O*_ and *R*_*L_C*_ show very similar long-term trends as daily mean *GPP*_*L_O*_ and *GPP*_*L_C*_, respectively (Fig 4d). Hence, daily mean *NEP*_*L_O*_ and *NEP*_*L_C*_ are substantially smaller than the other metabolic rates (Table 1), suggesting that lake gross primary production during daylight is sufficient to compensate lake respiration during day and night.

**Table 1:** Comparison of long-term mean lake metabolic rates estimated with the diel O_2_- and the diel CO_2_-technique and the influence of assumptions on vertical fluxes.

Transport processes considered and the effect of the net flux on concentration change	*$\frac{1}{A}\frac{\partial \left(A\cdot F\right)}{\partial z}$*	subscript label of metabolic rates	*diel CO*_*2*_*-technique*			*diel O*_*2*_*-technique*		
			lake gross production	lake respiration	lake netproduction	lake gross production	lake respiration	lake netproduction
			(mmol L^-1^d^-1^)			(mmol L^-1^ d^-1^)		
Fluxes at lake surface and at *Z*_*mix*_ are balanced (see Eq 23i)	0	*none*	0.028	0.029	-0.001	0.028	0.027	0.001
Gas exchange with the atmosphere (see Eq 23ii)	$\frac{A_{Surf}\cdot F_{atm}}{V_{Zmix}}$	*A*	0.028	0.031	-0.003	0.029	0.002	0.027
Gas exchange with the atmosphere, turbulent diffusion at *Z*_*mix*_ and mixed layer deepening (see Eq 23iii)	$\frac{A_{Surf}\cdot F_{atm}-A_{Zmix}\cdot F_{Zmix}}{V_{Zmix}}$	*F*				0.033	0.013	0.020
Gas exchange with the atmosphere and turbulent diffusion at *Z*_*mix*_ (see Eq 23iv)	$\frac{A_{Surf}\cdot F_{atm}-A_{Zmix}\cdot F_{turb}}{V_{Zmix}}$	*D*				0.029	0.003	0.026

Long-term means are calculated by averaging the daily mean metabolic rates of the 49 days for which daily mean metabolic rates were available from both techniques. The error of the mean metabolic rates is 0.002 mmol L^-1^ d^-1^ in case of the diel CO_2_-technique and 0.005 mmol L^-1^ d^-1^ in case of the diel O_2_-technique.

As O_2_ and CO_2_ are both oversaturated during most of the time (Fig 2d) the lake is emitting both gases, and the gas fluxes of both gases are therefore positive (Fig 2e). Consistently, including gas exchange with the atmosphere in the calculation of metabolic rates leads to lower estimates of the lake respiration *R*_*L_O*_,_*A*_ than *R*_*L_O*_ in case of the diel O_2_-technique (Fig 4c and 4e; Table 1), but to higher estimates of the lake respiration *R*_*L_C*_,_*A*_ than *R*_*L_C*_ in case of the diel CO_2_-technique (Fig 4b, Table 1). The difference between *R*_*L_C*,*A*_ and *R*_*L_C*_ in Fig 4b is particularly small because during the time period shown the oversaturation of CO_2_ is small (Fig 2d). However, the long-term average of the difference between *R*_*L_C*,*A*_ and *R*_*L_C*_ is also much smaller than that between *R*_*L_O*_ and *R*_*L_O*,*A*_ (Table 1), although the oversaturation of CO_2_ is on average 2.5 times larger than the oversaturation of O_2_ (average saturation of CO_2_ and O_2_ is 162% and 129%, respectively). Considering the fluxes due to turbulent mixing at the bottom of the mixed layer in addition to the surface flux results in respiration rates *R*_*L_O*,*D*_ that are slightly larger than *R*_*L_O*,*A*_ but still substantially smaller than *R*_*L_O*_ (Table 1). Respiration rates *R*_*L_O*,*F*_ estimated by considering atmospheric fluxes, fluxes due to turbulent diffusion and mixed layer deepening have values intermediate between *R*_*L_O*,*A*_ and *R*_*L_O*_ (Fig 4c and 4e, Table 1).

Estimates of lake gross primary production were comparatively insensitive to the assumptions on the transport processes, independent of whether the diel CO_2_- or the diel O_2_-technique was used (Fig 4b and 4c, respectively; Table 1). For all approaches considering different transport processes long-term averages of the lake gross primary production estimated from the diel CO_2_-technique had essentially the same values as those determined from the diel O_2_-technique (Fig 4d and 4e, Table 1).

The values of *GPP*_*L*_ were similar for diel O_2_- and diel CO_2_-technique and the different assumption on vertical transport, but *R*_*L*_ strongly depended on the assumptions on transport (Table 1). Hence, the estimates of *NEP*_*L*_ also strongly depended on the estimates of concentration changes due to transport processes (Table 1).

## Discussion

The CO_2_-optode provides reliable long-term data on *C*_*CO2*_ over several weeks at sub-hourly resolution, as is indicated by the good agreement between CO_2_ concentrations measured with the CO_2_-optode and the CO_2_-IRprobe, and by the long-term consistency of lake gross primary production estimated from the diel O_2_- and the diel CO_2_-technique (*GPP*_*L_O*_ and *GPP*_*L_C*_). Because CO_2_-optodes have a low power consumption they are ideally suited for long-term measurements of *C*_*CO2*_. Such data can be utilized to estimate metabolic rates using the diel CO_2_-technique and to determine CO_2_ fluxes from lakes based on direct measurements rather than indirect estimates of CO_2_.

Metabolic rates determined from the diel CO_2_-technique directly provide uptake and release of dissolved inorganic carbon due to production and respiration, whereas the diel O_2_-technique requires assumptions on the production and respiratory quotients if the contribution of metabolic transformations to the carbon balance is assessed. In alkaline Lake Illmensee (*pH* of ~8.5) the long-term averages of *GPP*_*L_C*_ and *GPP*_*L_O*_ agree well, suggesting that the production quotient *PQ* = *GPP*_*L_O*_ / *GPP*_*L_C*_ is close to one and thus within the range suggested by Oviatt et al. [34] and at the lower end for a typical algal cell [35]. However, according to measurements by Hanson et al. [2] in lakes with *pH* > 8 metabolic rates estimated with the diel O_2_-technique are substantially larger than estimates based on the diel change in CO_2_. This discrepancy can be explained by the dissociation of CO_2_ to bicarbonate and carbonate which substantially increases the temporal change in molar *C*_*DIC*_ compared to that of molar *C*_*CO2*_. In Lake Illmensee where *pH* ~ 8.5 the amplitude of the diel cycle of molar *C*_*DIC*_ is about five times larger than that of the diel cycle of molar *C*_*CO2*_ (Fig 3b and 3c). In contrast to the analysis of Hanson et al. [2], the diel CO_2_-technique employed in our study accounts for the dissociation of CO_2_ into different carbon species and estimates metabolic rates from the diel change in *C*_*DIC*_.

Similar to the system production quotient, the respiratory quotient *RQ = R*_*L_O*_ / *R*_*L_C*_ is close to one and thus within the range and close to the average value observed in estuarine mesocosm experiments [33]. However, the variability between days especially of *GPP*_*L_O*_ and *R*_*L_O*_ suggests considerable uncertainties in the estimates of the metabolic rates. Note that the production and respiratory quotients depend on the community of organisms responsible for the metabolic transformations and that the lake metabolic rates additionally depend on the exchange rates between the water column and the sediment (Eqs (14) and (17)).

The absolute values of *GPP*_*L_C*_ and *GPP*_*L_O*_ agree well with data on gross production measured with the diel O_2_-technique in other lakes (e.g., Lake Hampen, [3]; Lakes Peter and Paul, [21]). The pronounced daily cycle of *GPP*_*L_C*_ and *GPP*_*L_O*_ (Fig 4a) is consistent with the daily light cycle and light dependent production by phytoplankton. The ratios between lake gross production and lake respiration rate *GPP*_*L_C*_ / *R*_*L_C*_ and *GPP*_*L_O*_ / *R*_*L_O*,_ respectively, are close to one, which is consistent with the observations on metabolic ratios from several lakes [4,21]. Note that although the estimates of *GPP*_*L_C*_, *GPP*_*L_O*_, *R*_*L_C*_, and *R*_*L_O*_ do not include corrections for transport, they provide metabolic rates, metabolic ratios, and metabolic quotients *PQ* and *RQ* that are consistent with observations in other studies.

The estimates of lake gross primary production were not very sensitive to vertical fluxes due to transport processes (gas exchange, vertical mixing), which was in contrast to the estimates of lake respiration rates (Table 1). Because *GPP*_*L*_ is estimated from the difference between daylight *NEP*_*L*_ and average night-time *NEP*_*L*_, the estimates of *GPP*_*L*_ are only affected by the difference between the gradients of vertical fluxes during daytime and the average gradient of the vertical fluxes during night-time (for details see S6 appendix). Thus, if the gradients of the fluxes of O_2_, or of carbon respectively, do not change substantially between day and night, their effects on the estimates of lake gross primary production is small. In contrast to *GPP*_*L*_, estimates of lake respiration rates are affected directly by the average gradient of the vertical fluxes during night-time (Eqs (23) and (24); S6 appendix). Hence, if the gradients of the vertical fluxes have the same sign during day and night, as it was the case in our study, lake respiration rates are much more sensitive to the assumptions on the fluxes considered in the diel O_2_- and the diel CO_2_-techniques than lake gross primary production (Table 1).

Estimates of respiration rates based on the diel CO_2_-technique were much less sensitive to fluxes due to atmospheric gas exchange than estimates based on the diel O_2_-technique. As CO_2_ and O_2_ were nearly always oversaturated during day and night-time (Fig 2d) the fluxes due to gas exchange with the atmosphere are positive (Fig 2e). Hence, correcting estimates of metabolic rates for fluxes due to atmospheric gas exchange leads to increased respiration rates in the case of the diel CO_2_-technique and decreased respiration rates in case of the diel O_2_-technique (Table 1). However, the absolute change between *R*_*L_O*_ and *R*_*L_O*,*A*_ was much larger than that between *R*_*L_C*_ and *R*_*L_C*,*A*_ (Table 1), because the molar fluxes at the lake surface of CO_2_ were much smaller than those of O_2_ (Fig 2e). Even if the oversaturation of CO_2_ is larger than that of O_2_, the molar concentration *C*_*CO2*_ may be much smaller than *C*_*O2*_ (Fig 2b and 2c), since the molar atmospheric equilibrium concentration of CO_2_ is much smaller than that of O_2_ (e.g., at 20°C and local pressure (93600 Pa) *C*_*CO2*,*equ*_ = 0.014 mmol L^-1^ and *C*_*O2*,*equ*_ = 0.261 mmol L^-1^).

In general, the daily absolute change in the molar concentration difference between *in-situ* and atmospheric equilibrium concentration can be expected to be smaller for CO_2_ than for O_2_ (|*C*_*CO2*_-C_*CO2*,*equ*_| < |*C*_*O2*_-C_*O2*,*equ*_|). In alkaline Lake Illmensee much of the carbon taken up or released during metabolic processes is channeled to HCO_3_^-^ and CO_3_^2-^ and only about 20% of consumed or respired CO_2_ is visible in changes in *C*_*CO2*_ (Fig 3b and 3c). Thus only a fraction of the change in carbon associated with metabolic processes contributes to the gas exchange of CO_2_ with the atmosphere. In acidic lakes, the same production and respiration rates as in alkaline Lake Illmensee lead to substantially larger daily fluctuation in *C*_*CO2*_ [2] and thus may lead to larger effects of gas exchange on the estimated respiration rate than in alkaline Lake Illmensee. However, estimates of *R*_*L_C*_ and *R*_*L_O*_ can be expected to differ in their sensitivity to atmospheric gas exchange in many lakes because the atmospheric concentration of O_2_ is substantially larger than that of CO_2_ (20% O_2_ versus 0.04% CO_2_). Therefore, physical processes such as, e.g., introduction of gas-bubbles at the lake surface by breaking surface waves or changes in surface water temperature affecting solubility and thus atmospheric equilibrium concentrations alter molar under- or oversaturation of O_2_ much more than that of CO_2_.

Considering vertical transport due to turbulent diffusion and mixed layer deepening in the calculation of metabolic rates increases the estimated respiration rate *R*_*L_O*,*F*_ compared to the estimate *R*_*L_O*,*A*_ which considers only the gas exchange with the atmosphere (Table 1). Below the mixed surface layer *C*_*O2*_ typically increased with increasing water depth (Fig 2b, Fig Panels b and c in S2 appendix and Fig in S3 appendix). Turbulent diffusion and mixed layer deepening therefore cause a positive upwards flux of O_2_. Neglecting this flux leads to an underestimation to the lake respiration rate. The quantification of the effects of vertical mixing on the O_2_ budget is however rather crude. For example, the fluxes due to turbulent diffusion require values for turbulent diffusivities. These were determined from the empirical relations of [27] that however provide rather crude estimates of the turbulent diffusivities and are not validated for Lake Illmensee by independent means. Further, the 2 m spacing of the optodes does not provide a good vertical resolution of the O_2_ distribution.

Our calculations are based on the mass balance of O_2_ in the entire mixed surface layer and not in a shallower top layer of fixed vertical extension within the mixed surface layer as in Staehr et al. [3] and Obrador et al. [5]. The latter approach has the disadvantage that within the mixed surface layer vertical gradients of dissolved oxygen are very small and therefore cannot reliably be determined with O_2_-optodes. Furthermore, the empirical relations for *K*_*z*_ by Hondzo and Stefan [27], which were developed for stratified hypolimnia and not for mixed surface layers, provide unrealistically low diffusivities within the surface mixed layer.

The consequences of considering the turbulent flux of DIC and mixed layer deepening in the diel CO_2_-technique could not be assessed because of the lack of long-term data from which DIC could be determined at a second depth in addition to the time series at 1.7 m. However, the vertical profile of *C*_*DIC*_ calculated from the profiles of *C*_*CO2*_ and *T* measured on the 1^st^ of July and the profile of alkalinity measured on the 30^th^ of June, suggests that DIC increases with water depth (Fig in S3 appendix). In this case turbulent diffusion and mixed layer deepening leads to upward transport of carbon. A positive upwards flux of carbon implies that the lake respiration rates estimated with the diel CO_2_-technique considering only gas exchange with the atmosphere (*R*_*L_C*,*A*_) overestimate the true lake respiration rate.

The assumption that the gradient in the vertical fluxes of CO_2_ and of O_2_, respectively, is negligible leads to rather similar estimates of lake respiration rates with the diel CO_2_- and diel O_2_-techniques, i.e. *R*_*L_C*_ ≈*R*_*L_O*_ (Table 1). Consistently, considering only gas exchange with the atmosphere and neglecting turbulent transport from deeper layers leads to an increase in the discrepancies between the respiration rates, because the flux to the atmosphere is positive for both, CO_2_ and O_2_. Because *C*_*O2*_ and most likely also *C*_*DIC*_ increase below *Z*_*mix*_ with increasing water depth, also the vertical flux due to mixing is positive for O_2_ and DIC. In the diel O_2_-technique a positive upward flux of O_2_ into the observation layer implies lake respiration rates higher than *R*_*L_O*,*A*_ whereas in the diel CO_2_-technique a positive upward flux of DIC implies lake respiration rates lower than *R*_*L_C*,*A*_. Thus, in Lake Illmensee, the respiration rates *R*_*L_O*,*A*_ and *R*_*L_C*,*A*_ can be considered as the lower and upper bounds of the true lake respiration rates.

Lake respiration rates *R*_*L_O*_ estimated from *C*_*O2*_ measured at 3.2 m depth, *R*_*L_O*_ (3.2 m), and lake respiration rates estimated from *C*_*O2*_ measured at 1.2 m depth, *R*_*L_O*_ (1.2 m), show similar long-term development (Fig Panel b in S5 appendix) and differ on average by less than 15% (S5 appendix). The similarity in metabolic rates at the two depths is not surprising, because during most of the time, measurements from both depths were within the mixed surface layer. However, also during time periods when Z_mix_ < 3 m, e.g., between 7^th^ and 15^th^ of June, the estimates of *R*_*L_O*_ (3.2 m) and *R*_*L_O*_ (1.2 m) agreed rather well, except on the 10^th^ of June, when *R*_*L_O*_ (1.2 m) showed particularly strong deviations from the mean (Fig Panel b in S5 appendix). Considering the time period from the 7^th^ to the 15^th^ of June but excluding the 10^th^ of June, the average of *R*_*L_O*_ (3.2 m) (0.025 mmol L^-1^ d^-1^) agrees very well with the average of *R*_*L_O*_ (1.2 m) (0.023 mmol L^-1^ d^-1^), but the average of *R*_*L_O*,*A*_ (1.2 m) is negative (-0.005 mmol L^-1^). Note that the estimates of *R*_*L_O*_ neglect effects due to gradients in the vertical fluxes of oxygen whereas *R*_*L_O*,*A*_ considers gas exchange with the atmosphere but no other vertical fluxes. During the time period considered gas exchange with the atmosphere may influence the oxygen concentrations at 1.2 m but not at 3.2 m water depth because *Z*_*mix*_ < 3 m. The values of *R*_*L_O*,*A*_ (1.2 m) and *R*_*L_O*_ (3.2 m) agree well with each other but not with *R*_*L_O*,*A*_ (1.2 m) which assumes negative values that are conceptually impossible. These results suggest that considering gas exchange without including vertical transport into the mixed layer from below may result in a substantial underestimation of lake respiration rates and support the assumption that the net effect of all vertical fluxes is small.

Lake respiration rates not only include respiration in the open water but also oxygen consumption and carbon production at and within the sediments (Eqs (14) and (17)). Therefore, lake respiration rates not only depend on metabolic transformations but also on the exchange velocities between the sediment and the water column. The latter are controlled by the intensity of turbulence near the sediments and thus are affected by hydrodynamic processes that therefore indirectly influence the overall lake respiration rate.

In the surface mixed layer the aspect ratio between sediment area and water volume is small suggesting that the influence of fluxes into and from the sediments have only a small influence on the overall budget of O_2_ and CO_2_. However, the contribution of respiration within the sediments to overall oxygen consumption increases with water depth [36], because of the increase in the aspect ratio of sediment area to water volume. In the aphotic deep water zone of lakes oxygen depletion due to oxygen uptake by the sediments can be as large as or even larger than oxygen depletion in the open water column (e.g. [37]). Because in the deep water of lakes primary production may become very small due to light limitation NEP can be expected to become increasingly negative with increasing water depth leading to anoxic deep water bodies characterized by high concentrations of DIC (Fig Panels b, e, and f in S3 appendix). The deep water can thus act as a source of DIC for the surface layer, because the vertical gradient in *C*_*DIC*_ together with turbulent mixing leads to a positive vertical flux of DIC. If the conditions in the surface layer are at steady state this flux of DIC from below together with the effects of NEP on *C*_*DIC*_ are compensated by a CO_2_ flux to the atmosphere requiring oversaturation of CO_2_ in the surface mixed layer. Hence, the vertical flux of DIC from the anoxic deep water may explain the large oversaturation of CO_2_ at the beginning of the measuring time in early June (Fig 2d).

After the 7^th^ of June, primary production at intermediate water depth altered the vertical gradients of DIC and O_2_, as is indicated by the development of the oxygen maximum at ~7–8 m depth (Fig Panel b in S2 appendix and Fig Panel f in S3 appendix) and a local minimum in the vertical profile of *C*_*DIC*_ at this depth (Fig Panel e in S3 appendix). The decrease in CO_2_-oversaturation in the surface mixed layer during June and in July may thus be explained by reduced vertical fluxes of DIC. Analogously, the increase in the O_2_-oversaturation in the surface mixed layer after the 7^th^ of June was most likely caused by an increase in the vertical flux of O_2_ that was produced at intermediate depths.

The conditions under which it is advantageous to apply the diel CO_2_-technique and the limitations of this technique have been explored in a sensitivity study (S7 appendix). The main conclusions of this analysis can be summarized as follows. In lakes with pH < 8 the daily change in CO_2_, Δ*C*_*CO2*_, is an excellent estimator of the daily change in DIC, Δ*C*_*DIC*_, with Δ*C*_*CO2*_ typically being only ~10% smaller than Δ*C*_*DIC*_. However, in lakes with pH ≥ 8 the difference between Δ*C*_*CO2*_ and Δ*C*_*DIC*_ can be substantial and increases strongly with increasing pH, e.g., Δ*C*_*CO2*_ underestimates a Δ*C*_*DIC*_ of 0.02 mmol L^-1^ by more than 20% at pH = 8 and by a factor of ~5 at pH = 8.5 (Table A in S7 appendix). Hence, in alkaline lakes the assessment of daily changes in *C*_*DIC*_ from daily changes in *C*_*CO2*_ requires consideration of the carbonate balance.

If Δ*C*_*CO2*_ and *pH* and the balance of dissolved carbonates is used to estimate Δ*C*_*DIC*_, very small uncertainties in *pH* can introduce large errors in the estimate of Δ*C*_*DIC*_ especially if the water has pH ≥ 8, e.g., an uncertainty of 0.005 in *pH* may result in an overestimation of Δ*C*_*DIC*_ by a factor of two or more (Table B in S7 appendix), depending on the true Δ*C*_*DIC*_. Note that a systematic overestimation of pH has essentially no effect on the estimate of Δ*C*_*DIC*_.

The diel CO_2_-technique estimates *pH* from *C*_*CO2*_ and carbonate alkalinity and assumes that carbonate alkalinity is constant. In case alkalinity changes with time also carbonate alkalinity changes. The diel CO_2_-technique underestimates metabolic rates if Δ*C*_*DIC*_ due to metabolic processes and the change in carbonate alkalinity Δ*ALK*_*Carb*_ have the same sign and overestimates metabolic rates if Δ*C*_*DIC*_ due to metabolic processes and Δ*ALK*_*Carb*_ have opposite sign (Table C in S7 appendix). Changes in alkalinity caused by calcite precipitation or dissolution of solid carbonate have a smaller effect on the estimates of Δ*C*_*DIC*_ than the same alkalinity change caused by other ions (Table D in S7 appendix). However, because in many lakes alkalinity is dominated by bicarbonate and carbonate ions, calcite precipitation may be the primary cause of substantial changes in alkalinity. Note that a systematic underestimation or overestimation, respectively, of carbonate alkalinity has essentially no effect on the predicted Δ*C*_*DIC*_. Hence, slow changes in carbonate alkalinity over several days have only small effects on predicted daily changes in *C*_*DIC*_ and thus on the estimated metabolic rates. Further, using total alkalinity as measure of carbonate alkalinity has essentially no consequences for the estimated Δ*C*_*DIC*_.

The effects of changes in alkalinity on the estimates of metabolic rates could be avoided if high-precision *pH* measurements were available for the calculation of Δ*C*_*DIC*_. However, calcite precipitation and dissolution of solid carbonates not only affect alkalinity but also change *C*_*DIC*_. The diel CO_2_-technique treats all changes in *C*_*DIC*_ as consequence of metabolic transformations and transport processes and therefore cannot provide reliable results during time periods during which calcite precipitation and dissolution of solid carbonate result in large sinks or sources of DIC, respectively. However, if calcite precipitation or the dissolution of solid carbonates, respectively, occurs continuously during day and night, *GPP*_*L*_ estimated with the diel CO_2_-technique is much less sensitive to these processes than *R*_*L*_. This conclusion follows from the same argument that explained why *GPP*_*L*_ is less sensitive than *R*_*L*_ to transport processes if the gradient of the vertical flux has the same sign during day and night. In our study the time series of CO_2_ does not indicate sudden changes in CO_2_ which would accompany short-term events of calcite precipitation. The agreement between estimates of metabolic rates based on diel O_2_- and diel CO_2_-technique suggests that calcite precipitation was not a major factor in the balance of DIC but the same metabolic processes were responsible for the changes in DIC and O_2_.

The sensitivity study above suggests that it depends on the system whether metabolic rates can be reliably estimated with the diel CO_2_-technique or not. In shallow lakes and in littoral zones the dissolution of solid carbonates associated with the sediments may result in unreliable estimates of *R*_*L_C*_ but possibly do not substantially affect the reliability of estimates of *GPP*_*L_C*_. In the open water of deep lakes, the diel CO_2_-technique should provide reliable metabolic rates except during time periods of calcite precipitation. In small lakes with short residence times external loading of dissolved carbonates may affect reliability of the estimates of metabolic rates. Finally, in lakes with high alkalinity it is advantageous to base the diel CO_2_-technique on C_*CO2*_ and alkalinity rather than on *C*_*CO2*_ and pH or *C*_*CO2*_ alone.

## Conclusions

The diel CO_2_- and the diel O_2_-technique are complementary open-water methods for the estimation of metabolic rates in lakes. The diel CO_2_-technique has the advantage that it provides metabolic rates in terms of carbon produced or consumed and that it is less sensitive to gas exchange with the atmosphere. The assessment of metabolic rates with the diel CO_2_-technique is in principle not restricted to oxygenated regions of aquatic systems but can also be applied in anoxic waters, if instruments are available that can tolerate anoxic conditions. The diel CO_2_-technique could therefore be applied to investigate e.g. anaerobic methane oxidation which cannot be assessed with the diel O_2_-technique.

However, in contrast to the diel O_2_-technique, the diel CO_2_-technique requires additional measurements for the estimation of metabolic rates especially in alkaline lakes. In such lakes data on alkalinity or long-term *pH* measurements with sub-daily resolution must be available to determine the daily cycle of *C*_*DIC*_. In alkaline Lake Illmensee *C*_*DIC*_ estimated from *C*_*CO2*_ is very sensitive to *pH* (Fig in S4 appendix). Because sufficiently precise *pH* data with sub-daily temporal resolution over several weeks were not available, we utilized alkalinity to determine *C*_*DIC*_ from *C*_*CO2*_. In less alkaline lakes, e.g., in lakes with *pH* < 8 and an alkalinity that does not substantially exceed conditions at atmospheric equilibrium, time series of *C*_*CO2*_ may provide reliable estimates of *C*_*DIC*_.

The CO_2_-technique presented here treats alkalinity as an essentially conservative property because alkalinity is not affected by CO_2_ exchange with the atmosphere and changes due to production or respiration can be neglected. However, alkalinity may change due to several geochemical processes ([25]), e.g., calcite precipitation, nitrification and de-nitrification, inflow of water that has different alkalinity than the lake water, or vertical mixing, if alkalinity varies with water depth as in Lake Illmensee (Fig Panel c in S3 appendix). All these processes may increase the uncertainty of the metabolic rates estimated from the diel CO_2_-technique based on the combination of highly resolved time series of *C*_*CO2*_ with only a few alkalinity data.

Lake respiration rates are typically more difficult to estimate with the CO_2_- and O_2_-open-water techniques than gross primary production, because *R*_*L*_ directly depends on the night-time net source of DIC or O_2_, respectively, whereas the estimate of *GPP*_*L*_ depends on the difference between day-time and average night-time net source of DIC or O_2_, respectively. If the gradient in the vertical fluxes has the same sign during day and night, *R*_*L*_ is more sensitive to transport processes than gross primary production. Especially the assessment of fluxes due to mixing near the lake surface is demanding.

Comparison of metabolic rates estimated from diel CO_2_- and diel O_2_-technique can help to improve the reliability of conclusions on metabolic processes and the associated consumption or release of dissolved oxygen and carbon. For example, during periods of intense gas exchange with the atmosphere, *R*_*L_O*,*A*_ and *R*_*L_C*,*A*_ may provide the lower and upper bounds for the true respiration rate if O_2_ and CO_2_ are oversaturated. Time periods of calcite precipitation may be visible in systematic long-term shifts between lake respiration rates estimated with the diel CO_2_- and the diel O_2_-technique.

In this study the comparison of lake metabolic rates indicates that the production of dissolved oxygen and the uptake of dissolved inorganic carbon associated with gross primary production agree well in alkaline Lake Illmensee at a *pH* of ~8.5. Further, dissolved oxygen in the surface water is not only strongly affected by gas exchange with the atmosphere and metabolic processes within the surface layer but also by the transport of dissolved oxygen from deeper waters that originates from production in deep water. This suggest that lake respiration rates estimated from the oxygen balance within the surface layer considering gas-exchange with the atmosphere but neglecting turbulent transport within the water column may include parts of the net production from deeper layers that may have occurred at earlier times. In this case lake respiration rates are underestimated whereas primary gross production may not be affected if the oxygen flux from deeper layers does not vary within a day.

The long-term average of *NEP*_*L_O*_ and *NEP*_*L_C*_ were both close to zero. Nevertheless, CO_2_ and O_2_ were oversaturated with respect to atmospheric equilibrium and the system was emitting both gases at the same time. Apparently, O_2_ emissions were not dominated by the current metabolism in the surface mixed layer but mainly linked to vertical transport of oxygen from an oxygen maximum at ~7–8 m water depth that must have been the result of net oxygen production at this depth most likely during the build-up of a phytoplankton layer in the deep water. Similarly, the CO_2_ emissions were not linked directly to the *NEP*_*L_C*_ estimated from the *C*_*DIC*_ in the surface water but resulted from vertical transport of DIC that had been released in deeper waters and in the anoxic sediments. The comparison of lake metabolic rates estimated from the diel CO_2_- and the O_2_-technique demonstrates that estimates of *NEP* based on measurement in the surface water do not reliably indicate system heterotrophy or autotrophy even if the data cover time periods of two months indicating the need for seasonal vertically-resolved carbon and oxygen-based estimates of metabolic rates.

## Supporting Information

S1 Appendix Background data on wind speed, water column characteristics and transport. (PDF)

S2 Appendix Long-term development of temperature stratification and the vertical distribution of dissolved oxygen. (PDF)

S3 Appendix Vertical distribution of *pCO*_*2*_, temperature, alkalinity, *pH*, *C*_*DIC*_, *C*_*O2*_ and *Chl*_*a*_. (PDF)

S4 Appendix Sensitivity of the concentration of DIC to daily changes in *pH*. (PDF)

S5 Appendix Comparison of metabolic rates obtained using two different approaches to estimate night-time respiration and of metabolic rates determined from *C*_*O2*_ measured at 1.2 m and 3.2 m water depth. (PDF)

S6 Appendix Estimates of lake gross primary production *GPP*_*L*_ are less sensitive to vertical transport than estimates of lake respiration rates *R*_*L*_ obtained from the diel CO_2_-technique: Mathematical illustration. (PDF)

S7 Appendix Metabolic rates estimated with CO_2_-technique: Sensitivity to pH and alkalinity. (PDF)

S8 Appendix Compilation the main equations of the CO_2_- and the O_2_-technique. (PDF)

S9 Appendix Compilation of the empirical relations used in this study. (PDF)
