# Supplementary material for: Lake Metabolism: Comparison of Lake Metabolic Rates Estimated from a Diel CO2- and the Common Diel O2-Technique
Source: PLoS One. 2016 Dec 21;11(12):e0168393. doi: 10.1371/journal.pone.0168393 (PMC5176309; doi:10.1371/journal.pone.0168393)
Supplement: S1 Appendix — (PDF) [file pone.0168393.s001.pdf]

S1 Appendix: Background data on wind speed, water column characteristics and transport

Wind speed 10 m above the water surface (Fig Panel a) was determined from measurements collected on the lake 1.5 m above the lake surface assuming a logarithmic boundary layer. Thereby we considered a wind speed dependent drag coefficients C_{10} using the relation of Wu [1] and assuming $C_{10} \geq 10^{-3}$. The water temperatures measured at the water depths of the O_2-optodes (1.2 and 3.2 m) and at the depth of the CO_2-optodes (1.7 m) are shown in Fig Panel b.

Mixed layer depth Z_{mix} (Fig Panel c) was determined from temperature data collected with thermistors moored with a vertical resolution of 1 m. (see also Fig Panel a in S2 Appendix). Water density was calculated from these temperatures using the equation of state by Chen and Millero [2]. Vertical density profiles were obtained by linear interpolation of density whereby the uppermost density is available from the uppermost thermistor moored at 1.2 m depth. Z_{mix} was defined as the depth at which density is 0.1 kg m^{-3} larger than the density at 1.2 m depth. Using a prescribed density difference with respect to the uppermost available density value instead of a prescribed local density gradient as in Staehr et al. [3] relates the surface mixed layer depth to the energy input into the surface layer to overcome potential energy rather than to local density gradients at larger depth.

Turbulent diffusivities K_z were estimated using the empirical equations of Hondzo and Stefan [4] as suggested by Staehr et al. [3]. These empirical relations have been developed to provide rough estimates of turbulent diffusivities in the hypolimnion of small lakes. Note that the empirical relations in Staehr et al. [3] do not exactly correspond to the original relations and lead to K_z that are overestimated by an order of magnitude compared to the relations by Hondzo and Stefan [4]. The Brunt-Väisälä frequency required for the estimation of K_z was determined from the temperature data measured with the thermistor chain. K_z at the bottom boundary of the mixed layer, i.e. at Z_{mix}, was determined by linear interpolation (Fig Panel d).

Water temperatures at the depth of the uppermost O_2-optode moored at 1.2 m depth and at the depth of the CO_2-optode moored at 1.7 m depth agree rather well during the entire measuring period (Fig Panel b). This indicates that both optodes were located within the surface mixed layer, which is also confirmed by the estimated Z_{mix} of typically 2 m or larger (Fig Panel c). Between the 7th and the 15th of June and the 16th and 21st of July temperatures at the location of the second O_2-optode moored at 3.2 m water depth were substantially colder than the temperatures at the depth of the other optodes indicating a mixed surface layer shallower than 3

m. During the same time periods Z_{mix} is smaller than 3 m (Fig Panel c), suggesting that the water column is stratified between 1.2 m and 3.2 m water depth.

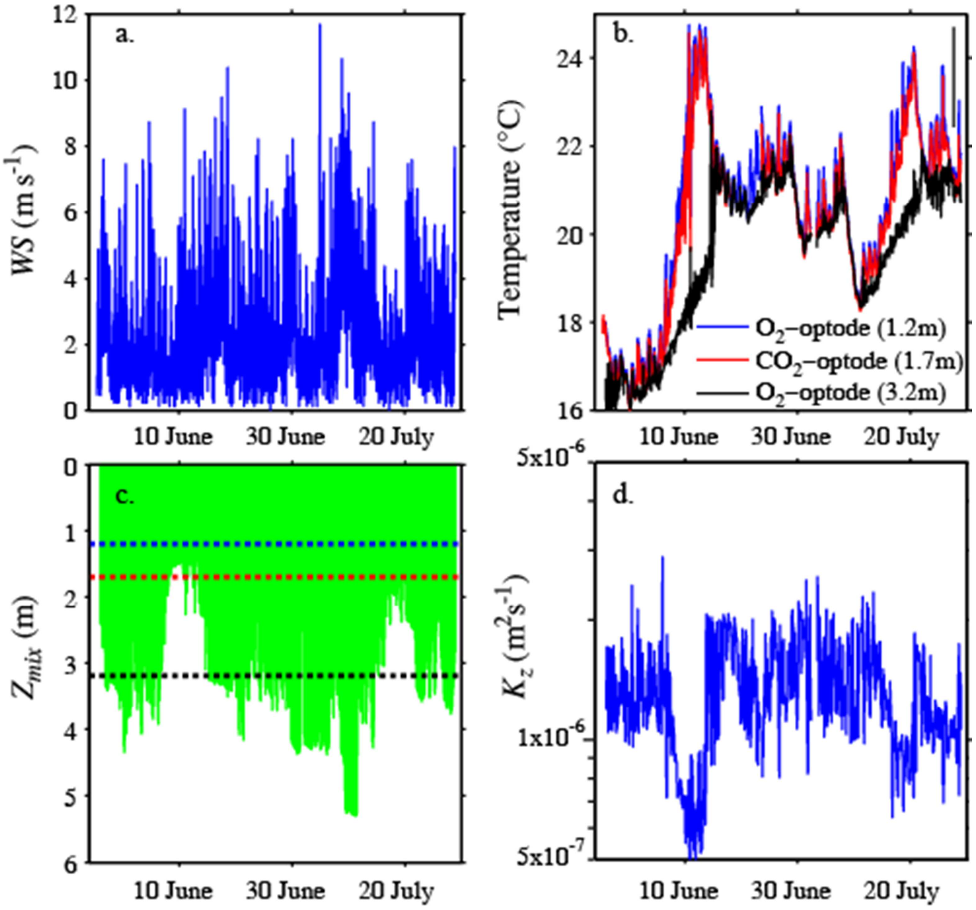

Fig. Background data on wind speed, water column characteristics and turbulent diffusivities.
(a) Wind speed, (b) Temperature at the water depth of the CO_2-optode and the two uppermost O_2-optodes. (c) Surface mixed layer depth Z_{mix}; (d) Turbulent diffusion coefficient K_z at Z_{mix}. Dashed lines in (c) indicate the water depth of the two O_2-optodes (blue and black) and of the CO_2-optode (red).

References

1. Wu J. Wind-stress coefficients over sea surface near neutral conditions - A revisit. J Phys Oceanogr. 1980;10(5):727–40.
2. Chen C-TA, Millero FJ. Precise thermodynamic properties for natural waters covering only the limnological range. Limnol Oceanogr. 1986;31(3):657–62.
3. Staehr PA, Christensen JPA, Batt R, Read J. Ecosystem metabolism in a stratified lake. Limnol Oceanogr [Internet]. 2012 [cited 2014 Oct 23];57(5):1317–30. Available from: http://www.aslo.org/lo/toc/vol_57/issue_5/1317.html
4. Hondzo M, Stefan HG. Lake water temperature simulation model. J Hydraul Eng. 1993;119:1251–73.
