# Supplementary material for: Lake Metabolism: Comparison of Lake Metabolic Rates Estimated from a Diel CO2- and the Common Diel O2-Technique
Source: PLoS One. 2016 Dec 21;11(12):e0168393. doi: 10.1371/journal.pone.0168393 (PMC5176309; doi:10.1371/journal.pone.0168393)
Supplement: S2 Appendix — (PDF) [file pone.0168393.s002.pdf]

S2 Appendix: Long-term development of temperature stratification and the vertical distribution of dissolved oxygen

The development of temperature stratification and of the vertical distribution of dissolved oxygen is shown in Fig Panels a,b. During the entire time period the water column was stratified as is indicated by strong temperature gradients in the upper meters of the water column (Fig Panel a). Together with the increase of the surface temperature shortly before the 10th of June, deep-water oxygen concentrations increased substantially and a pronounced oxygen maximum at ~ 7m water depth was present from the 7th of June onwards (Fig Panel b). Oxygen concentrations at 5.2 m depth were larger than at shallower depth during most of the time (Fig Panel c). The negative gradients in dissolved oxygen from ~5 m depth towards the surface suggest a positive vertical flux of oxygen due to mixing processes. The box-car filtered time series of C_{O2} presented in Fig Panel c were obtained by applying a 6 h running mean to the O_2-data from the three uppermost optodes that measured at 1.2 m, 3.2 m and 5.2 m water depth. The time series show daily oscillations with increasing C_{O2} during the daytime and decreasing C_{O2} during night-time indicating positive net-production during the day and respirational losses during the night.

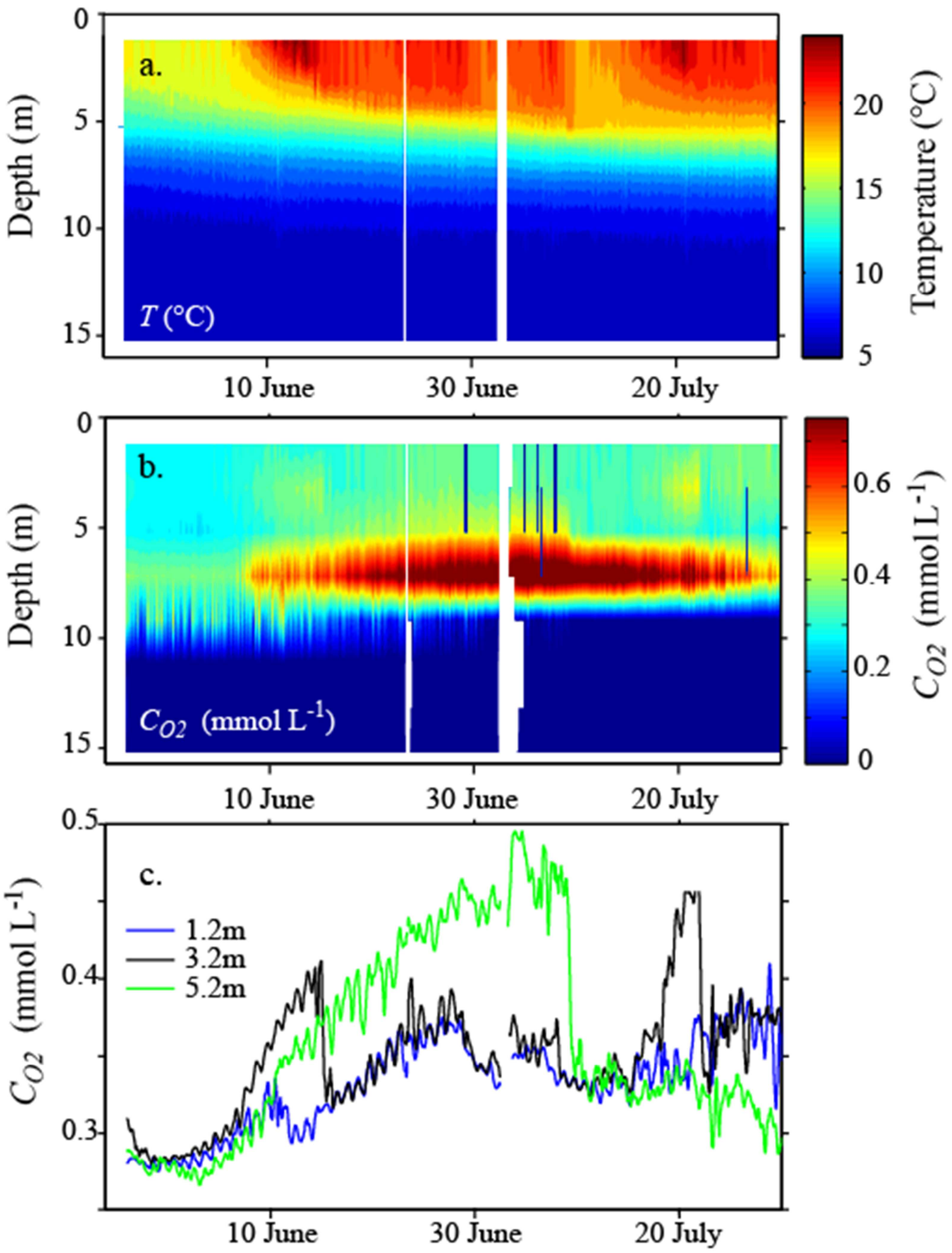

Fig. Temperature stratification and the distribution of the dissolved oxygen concentration.
(a) Temperature stratification of Lake Illmensee during the measuring period indicating a strong temperature gradient in the upper meters of the water column. (b) Distribution of C_{O2} indicating that after that the 7th of June a pronounced oxygen maximum developed at ~ 7m depth. (c) Time series of C_{O2} measured with the uppermost 3 optodes at 1.2 m, 3.2 m and 5.2 m water depth. Data were box-car filtered using a 6 hour running mean. Daily cycles in the oxygen concentrations indicate positive net-production during day time and respiration during night time. Between the 10th of June and the 10th of July oxygen concentrations below the mixed layer are substantially higher than within the mixed layer.
