# Supplementary material for: Lake Metabolism: Comparison of Lake Metabolic Rates Estimated from a Diel CO2- and the Common Diel O2-Technique
Source: PLoS One. 2016 Dec 21;11(12):e0168393. doi: 10.1371/journal.pone.0168393 (PMC5176309; doi:10.1371/journal.pone.0168393)
Supplement: S3 Appendix — (PDF) [file pone.0168393.s003.pdf]

**S3 Appendix: Vertical distribution of $pCO_2$, temperature, alkalinity, $pH$, $C_{DIC}$, $C_{O2}$ and $Chl_a$**

Vertical profiles of $pCO_2$, temperature, $pH$, $C_{O2}$ and $Chl_a$ measured on the 1st of July are depicted in Fig. Water samples for the alkalinity profile (Fig Panel c) were collected on the 30th of June. The $pCO_2$ data were measured by lowering the $CO_2$-IRprobe to predefined depths at which it was positioned for 20 min to allow adjustment to the ambient concentration. Completion of the profile lasted from 12 am to 3 pm. The profile of dissolved oxygen was measured with the multi-parameter CTD (RBR) at 1 pm. $Chl_a$ was measured with a multi-spectral fluorescence probe (Moldaenke FluoroProbe). Additionally, profiles of modelled pH and modelled $C_{DIC}$ were determined from *pCO2*, alkalinity and temperature (circles in Fig Panels d,e).

At about 7.5 to 8.5 m water depth $C_{O2}$ exceeds the measuring range of the $O_2$-optode of the multi-parameter CTD (Fig Panel f). The oxygen maximum is at a slightly larger depth than in Fig Panel b in S2 Appendix. Note, that Fig Panel b in S2 Appendix is based on a linear interpolation of data from $O_2$-optodes mounted with a 2 m vertical spacing. In the depth range of the oxygen maximum, $O_2$-optodes were mounted at 7.2 m and 9.2m depth. The oxygen peak coincides with a strong sharp peak in $Chl_a$ (Fig Panels f,g). The spectrum of the fluorescence signal and water sample analysis suggests that the deep chlorophyll maximum is caused by a high abundance of *P. rubescens*. Below 10 m the water column becomes anoxic which coincides with large values of $pCO_2$, alkalinity and modelled $C_{DIC}$. Because of the limited temporal response of the $O_2$-optode the vertical transition to the anoxic zone is somewhat smoothed in the vertical profile shown. The moored $O_2$-optodes indicate that already at 9.2 m water depth $C_{O2}$ are below 0.02 mmol $L^{-1}$ (Fig Panel b in S2 Appendix).

Alkalinity and $C_{DIC}$ increase with increasing depth whereas $pH$ decreases with increasing depth. The values of $pH$ predicted from $pCO_2$ and alkalinity agree well with the measured $pH$ except at 8 m water depth. At 8 m water depth measured alkalinity and $pH$ substantially deviate from their general vertical trend. These deviations may be related to the high abundance of *P. rubescens* and the high $C_{O2}$ at 8 m water depth.

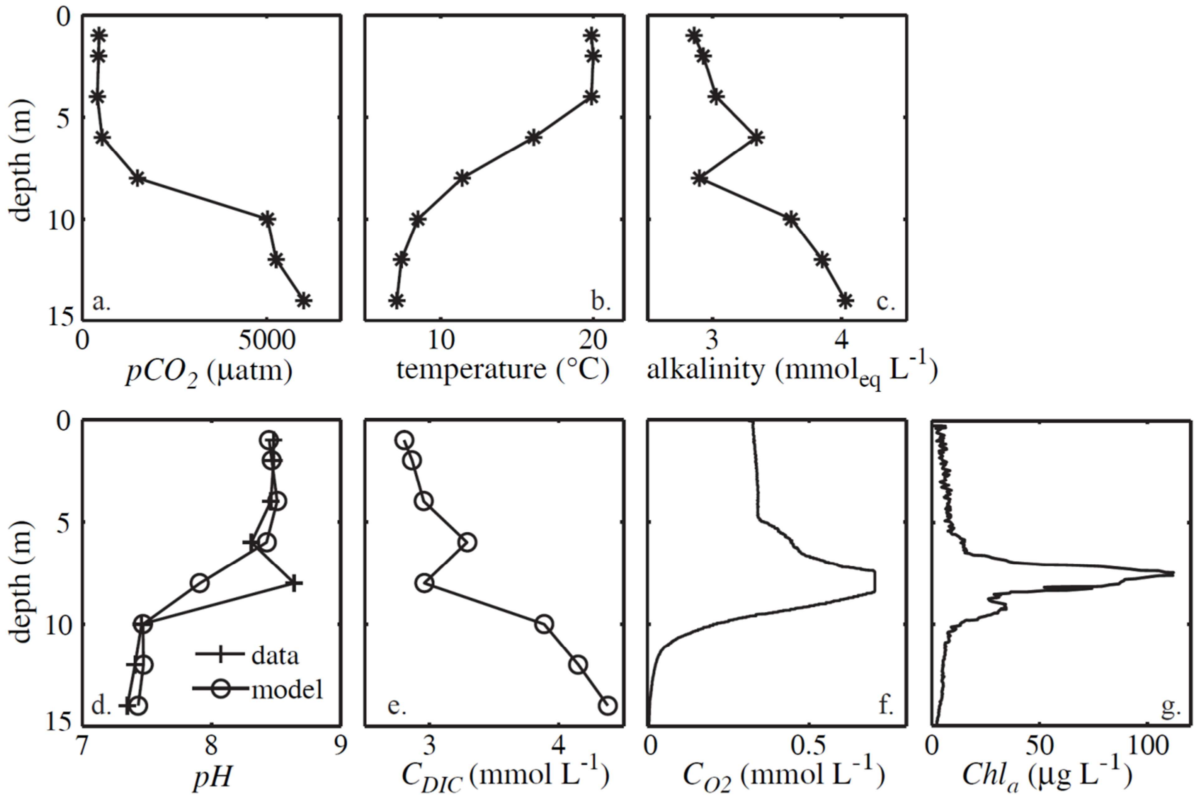


**Fig. Vertical distribution of $pCO_2$, temperature, alkalinity, pH, $C_{DIC}$, ,$C_{O2}$ and $Chl_a$**
Measured properties are characterized by *, + and black solid lines. $C_{DIC}$ and *pH* derived from $pCO_2$, alkalinity and temperature are depicted by open circles connected by black lines. The symbols * and + indicate the depth at which water samples were collected. $C_{O2}$ was measured with an $O_2$-optode attached to a CTD probe. $Chl_a$ was measured with a multi-spectral fluorescence probe.
