# Supplementary material for: Lake Metabolism: Comparison of Lake Metabolic Rates Estimated from a Diel CO2- and the Common Diel O2-Technique
Source: PLoS One. 2016 Dec 21;11(12):e0168393. doi: 10.1371/journal.pone.0168393 (PMC5176309; doi:10.1371/journal.pone.0168393)
Supplement: S4 Appendix — (PDF) [file pone.0168393.s004.pdf]

S4 Appendix: Sensitivity of the concentration of DIC to daily changes in *pH*

A time series of *pH* was determined from time series of pCO_2 and temperature assuming a time constant alkalinity of 2.95 $mmol_{eq}$ L^{-1} (Fig 3a). C_{DIC} calculated from the time series of *pH*, pCO_2 and temperature is shown in Fig 3a of the main manuscript and in Fig (black line). During the time period from the 23rd June to the 3rd July the average *pH* was 8.45. Using a time constant *pH* = 8.45 and the measured time series of pCO_2 and temperature leads to a time series of C_{DIC} (Fig, blue line) that shows pronounced daily cycles that have similar shape but more than 20 times larger amplitudes than the daily cycles of C_{DIC} estimated from the time variable *pH* (Fig, black line).

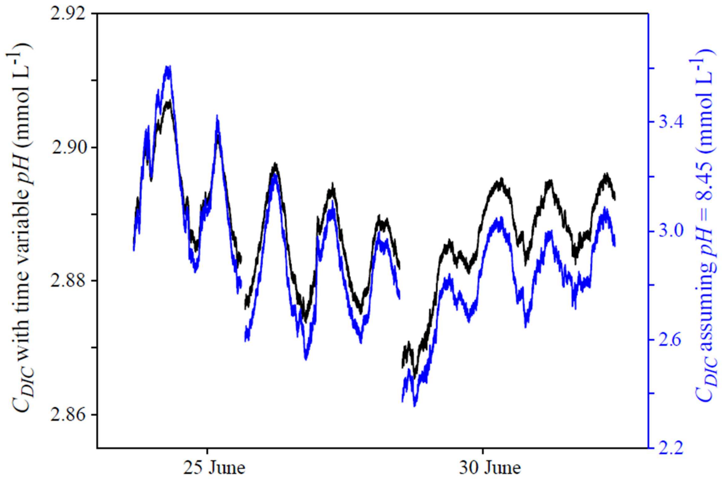

Fig. Sensitivity of C_{DIC} to daily changes in *pH*.
C_{DIC} calculated from the time variable *pH* depicted in Fig 3a of the main manuscript (black line) and C_{DIC} calculated using the mean *pH* during the time period shown (*pH* = 8.45) (blue line) show daily cycles that are similar in shape but have substantially different amplitudes. Note the different scaling of the left and right axis.
