# Supplementary material for: Lake Metabolism: Comparison of Lake Metabolic Rates Estimated from a Diel CO2- and the Common Diel O2-Technique
Source: PLoS One. 2016 Dec 21;11(12):e0168393. doi: 10.1371/journal.pone.0168393 (PMC5176309; doi:10.1371/journal.pone.0168393)
Supplement: S5 Appendix — (PDF) [file pone.0168393.s005.pdf]

S5 Appendix: Comparison of metabolic rates obtained using two different approaches to estimate night-time respiration and of metabolic rates determined from C_{O2} measured at 1.2 m and 3.2 m water depth

Lake respiration rates R_L can be estimated from the mean net production during night-time (Eq. (24)) or from linear regression of the temporal development of net production during night-time (Eq. (25)). Using as an example the diel CO_2-technique applied to the data from Lake Illmensee, Fig Panel a illustrates that the metabolic rates determined with the two approaches to estimate night-time respiration rates agree rather well. The metabolic rates obtained by using the mean net production during night-time for the estimation of R_L are summarized in the Table. Metabolic rates obtained by using linear regression for the estimation of R_L are summarized in Table 1 of the main manuscript.

Table. Lake metabolic rates obtained from the diel CO_2-technique by estimating lake respiration rate from the mean lake net production at night.

GPP_{L_C} (mmol $L^{-1}d^{-1}$)	0.026	$GPP_{L\_C,A}$ (mmol $L^{-1}d^{-1}$)	0.026
R_{L_C} (mmol L^{-1} d^{-1})	0.026	$R_{L\_C,A}$ (mmol L^{-1} d^{-1})	0.029
NEP_{L_C} (mmol $L^{-1}d^{-1}$)	0.000	$NEP_{L\_C,A}$ (mmol L^{-1} d^{-1})	-0.003

Application of the diel O_2-technique to the data on dissolved oxygen from 1.2 m and 3.2 m water depth suggests that daily mean lake metabolic rates GPP_{L_O} and R_{L_O} are similar at 1.2 m and 3.2 m water depth and that average NEP_{L_O} is close to zero at both depths (Fig Panel b). Averaging the daily mean metabolic rates over all days for which daily mean metabolic rates are available from 1.2 m and 3.2 m depth provide mean values for GPP_{L_O} of 0.030 mmol L^{-1} d^{-1} at 1.2 m and 0.034 mmol L^{-1} d^{-1} at 3.2 m, R_{L_O} of 0.028 mmol L^{-1} d^{-1} at 1.2 m and 0.032 mmol L^{-1} d^{-1} at 3.2 m, and NEP_{L_O} of 0.002 mmol L^{-1} d^{-1} at 1.2 m and at 3.2 m depth. The similar values of the metabolic rates at the two depths are comprehensible, because during most of the time, measurements from both depths were within the mixed surface layer. However, e.g. between the 7th and the 15th June the mixed layer depth was less than 3 m (Fig in S1 Appendix). Nevertheless,

the time series of GPP_{L_O} and R_{L_O} from 1.2 m and 3.2 m depths were still similar, except on the 10th of June when GPP_{L_O} and R_{L_O} at 1.2m depth showed particularly strong deviations from the mean (Fig Panel b). Note that the calculations of the metabolic rates GPP_{L_O} and R_{L_O} assume that the net fluxes due to vertical diffusive transport have negligible effects on the dissolved oxygen concentrations. Between the 11th and 15th of June the average respiration at 1.2 m depth estimated by considering atmospheric gas exchange but no other vertical fluxes of O_2 ($R_{L\_O,A}$ in Fig 5c) is negative ($R_{L\_O,A}$ (1.2 m) = -0.023 mmol L^{-1} d^{-1}), whereas the average R_{L_O} at 3.2 m depth is positive (R_{L_O} (3.2m) = 0.025 mmol L^{-1} d^{-1}) and close to the long term mean. During this specific time period considered, the mixed layer depth is shallower than 3 m. Therefore, estimates of respiration based on the data from 3.2 m water depths cannot be affected by gas exchange with the atmosphere. The conceptually impossible negative values of $R_{L\_O,A}$ (1.2 m) and the reasonable values of R_{L_O} (3.2 m) suggest that considering only atmospheric gas exchange without including vertical transport into the mixed layer from below may result in a substantial underestimation of respiration.

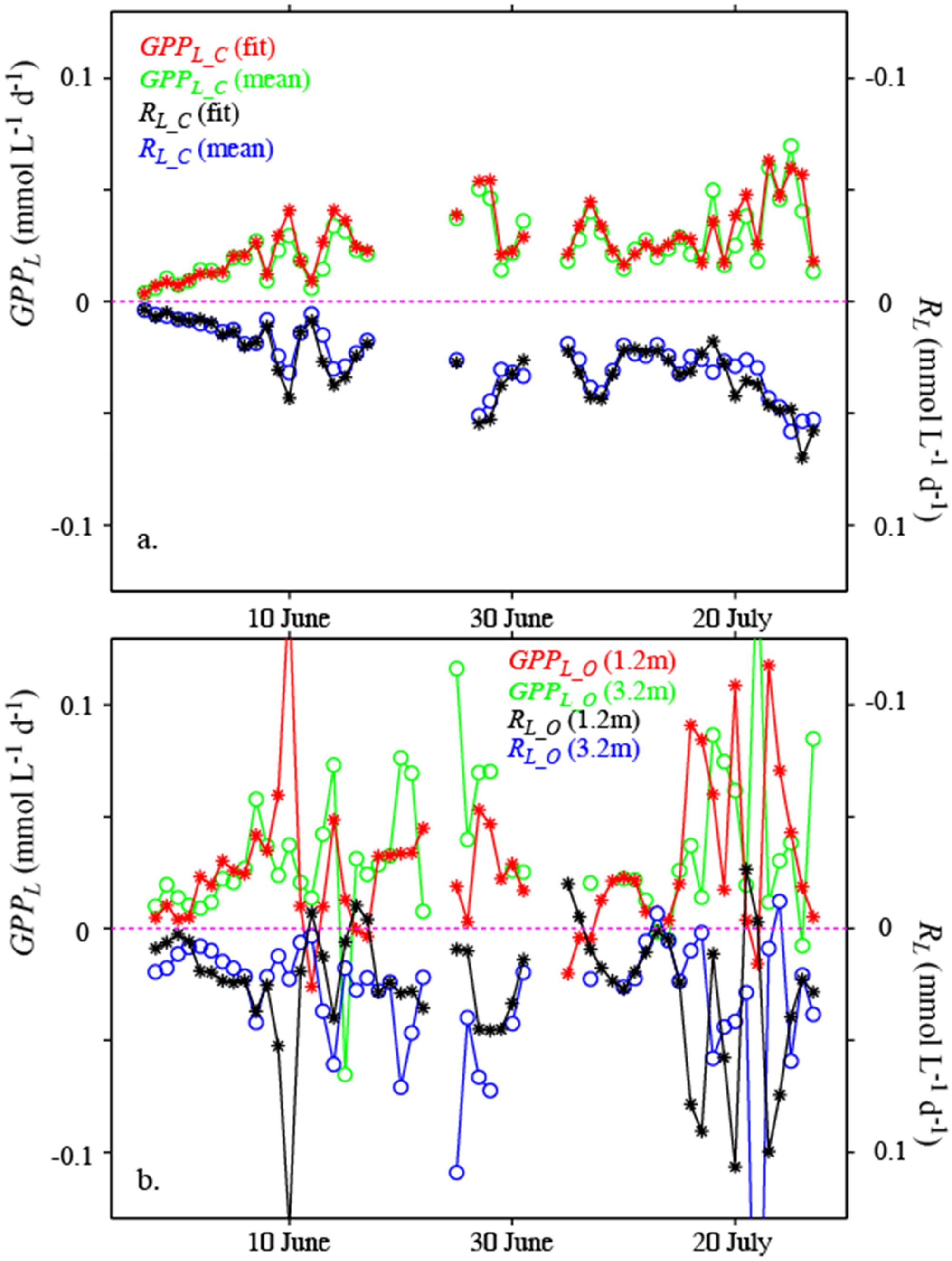

Fig. Comparison of lake metabolic rates.
(a) Metabolic rates obtained by linear regression (fit) and by averaging (mean) of night-time NEP_L. (b) Metabolic rates determined by the diel O_2-technique applied to oxygen data measured at 1.2 and 3.2 m water depth.
