# Supplementary material for: Lake Metabolism: Comparison of Lake Metabolic Rates Estimated from a Diel CO2- and the Common Diel O2-Technique
Source: PLoS One. 2016 Dec 21;11(12):e0168393. doi: 10.1371/journal.pone.0168393 (PMC5176309; doi:10.1371/journal.pone.0168393)
Supplement: S6 Appendix — (PDF) [file pone.0168393.s006.pdf]

S6 Appendix: Estimates of lake gross primary production GPP_L are less sensitive to gradients in vertical fluxes than estimates of lake respiration rates R_L: Mathematical illustration

The conclusion that estimates of lake gross primary production GPP_L are less sensitive to gradients in vertical fluxes than estimates of lake respiration rates R_L applies to both techniques, the diel O_2-technique and the diel CO_2-technique.

In the diel O_2-technique lake gross primary production and lake respiration rates are given by:

$$
\begin{aligned}
GPP_{L\_O}(t) - R_{L\_O,night} &= \frac{\partial C_{O2}(t)}{\partial t} + \frac{1}{A}\frac{\partial\left(A\cdot F_{O2}(t)\right)}{\partial z} \\
R_{L\_O,night} &= -mean\left(\frac{\partial C_{O2}(t_{night})}{\partial t} + \frac{1}{A}\frac{\partial\left(A\cdot F_{O2}(t_{night})\right)}{\partial z}\right) \\
GPP_{L\_O}(t) &= \frac{\partial C_{O2}(t)}{\partial t} - mean\left(\frac{\partial C_{O2}(t_{night})}{\partial t}\right) \\
&\quad + \frac{1}{A}\frac{\partial\left(A\cdot F_{O2}(t)\right)}{\partial z} - mean\left(\frac{1}{A}\frac{\partial\left(A\cdot F_{O2}(t_{night})\right)}{\partial z}\right)
\end{aligned}
\qquad \text{(S1)}
$$

whereby the respiration rate was estimated from the mean NEP_{L_O} at night.

If the vertical gradients of the vertical fluxes at different times during a day do not differ much from the mean gradient of the vertical fluxes during night-time:

$$
\frac{\partial\left(A\cdot F_{O2}(t)\right)}{\partial z} \approx mean\left(\frac{\partial\left(A\cdot F_{O2}(t_{night})\right)}{\partial z}\right) \qquad \text{(S2)}
$$

In this case neglecting gradients in the vertical fluxes does not affect estimates of lake gross primary production as can be seen by combining Eqs. (1) and (2):

$$
GPP_{L\_O}(t) = -\frac{\partial C_{O2}(t)}{\partial t} + mean\left(\frac{\partial C_{O2}(t_{night})}{\partial t}\right) \qquad \text{(S3)}
$$

In contrast to lake gross primary production, the lake respiration rate directly depends on the mean of the gradient in the oxygen fluxes during night-time (see Eq. (S1)) and is therefore much more sensitive to the gradients of vertical oxygen fluxes during night-time. Note, that only the gradient in the fluxes and not the fluxes themselves effect the estimated metabolic rates.

An analogous argument can be derived for GPP_{L_C} and R_{L_C} estimated from the diel CO_2-technique:

$$\begin{aligned} GPP_{L\_C}(t) - R_{L\_C,night} &= -\frac{\partial C_{DIC}(t)}{\partial t} - \frac{1}{A}\frac{\partial\left(A\cdot F_{DIC}(t)\right)}{\partial z} \\ R_{L\_C,night} &= mean\left(\frac{\partial C_{DIC}(t_{night})}{\partial t} + \frac{1}{A}\frac{\partial\left(A\cdot F_{DIC}(t_{night})\right)}{\partial z}\right) \\ GPP_{L\_C}(t) &= -\frac{\partial C_{DIC}(t)}{\partial t} + mean\left(\frac{\partial C_{DIC}(t_{night})}{\partial t}\right) \\ &\quad -\frac{1}{A}\frac{\partial\left(A\cdot F_{DIC}(t)\right)}{\partial z} + mean\left(\frac{1}{A}\frac{\partial\left(A\cdot F_{DIC}(t_{night})\right)}{\partial z}\right) \end{aligned} \quad \text{(S4)}$$

If the gradients of the vertical fluxes at different times during a day do not differ much from the mean gradient of the vertical fluxes during night-time:

$$\frac{\partial\left(A\cdot F_{DIC}(t)\right)}{\partial z} \approx mean\left(\frac{\partial\left(A\cdot F_{DIC}(t_{night})\right)}{\partial z}\right) \quad \text{(S5)}$$

In this case neglecting gradients of carbon fluxes does not affect estimates of lake gross primary production as can be seen by combining Eqs. (S4) and (S5):

$$GPP_{L\_C}(t) = -\frac{\partial C_{DIC}(t)}{\partial t} + mean\left(\frac{\partial C_{DIC}(t_{night})}{\partial t}\right) \quad \text{(S6)}$$

Note that the gradients of the vertical fluxes during night time have opposite sign in the calculation of R_{L_O} and R_{L_C}. Also the deviation between the gradients in the fluxes during day time and the night time mean of the gradient of the fluxes have opposite sign in the calculation of GPP_{L_O} and GPP_{L_C}. Hence, if concentration gradients of C_{O2} and C_{DIC} have the same sign

changes in diffusivities have opposite effects on the metabolic rates. Further, if O_2 and CO_2 are both oversaturated, sub-daily fluctuations in wind speed have opposite effects on the estimates GPP_{L_O} and GPP_{L_C}.
