# Supplementary material for: Lake Metabolism: Comparison of Lake Metabolic Rates Estimated from a Diel CO2- and the Common Diel O2-Technique
Source: PLoS One. 2016 Dec 21;11(12):e0168393. doi: 10.1371/journal.pone.0168393 (PMC5176309; doi:10.1371/journal.pone.0168393)
Supplement: S7 Appendix — (PDF) [file pone.0168393.s007.pdf]

S7 Appendix: Metabolic rates estimated with CO_2-technique: Sensitivity to pH and alkalinity

The diel CO_2-technique determines metabolic rates from sub-daily changes in C_{DIC}, ΔC_{DIC}. The rate of change in C_{DIC} is equal to *NEP* if metabolic processes are the only source or sink of DIC. However, in the diel CO_2-technique C_{DIC} is not measured but estimated from CO_2 data. This section investigates under which circumstances it is advantageous to estimate ΔC_{DIC} directly as ΔC_{CO2}, or to calculate ΔC_{DIC} considering the carbonate system utilizing ΔC_{CO2} and pH or alternatively ΔC_{CO2} and alkalinity.

Assuming similar conditions as in Lake Illmensee (T = 20°C, S=0.25 g kg^{-1} and $pCO2_{atm}$ = 365 µatm) we have calculated ALK_{Carb} and C_{DIC} in atmospheric equilibrium considering waters with different pH (6.0, 7.0, 8.0, 8.3, and 8.5). ALK_{Carb} and C_{DIC} increase strongly with increasing pH and are two orders of magnitude larger at pH 8.5 than at pH 6.0 (Table A).

Table A. Conditions at atmospheric equilibrium and the implication of an increase in DIC.
In atmospheric equilibrium ALK_{Carb} and C_{DIC} differ substantially in lakes with different pH. The same increase in C_{DIC} due to respiration leads to an increase in C_{CO2} that strongly depends on the alkalinity in the system.

pH	ALK_{Carb} mmol$_{eq}$ L^{-1}	C_{DIC} mmol L^{-1}	ΔC_{DIC} mmol L^{-1}	ΔC_{CO2} mmol L^{-1}	ΔC_{CO2}/ ΔC_{DIC} %
6.0	0.007	0.022	0.020	0.019	95
7.0	0.078	0.093	0.020	0.020	99
8.0	0.797	0.803	0.020	0.016	79
8.3	1.620	1.603	0.020	0.009	44
8.5	2.623	2.560	0.020	0.004	21

Estimates of ΔC_{DIC} utilizing ΔC_{CO2} measurements

If 0.020 mmol L^{-1} DIC is added to the system, i.e ΔC_{DIC} = 0.020 mmol L^{-1}, and ALK_{Carb} remains constant, the equilibration of the carbonate system causes a change in C_{CO2} that strongly depends on the initial pH of the system and is always smaller than ΔC_{DIC} (Table A). The chosen value of ΔC_{DIC} = 0.02 mmol L^{-1} is within the range of the typical daily change in C_{DIC} in Lake Illmensee (Fig. 3a).

The results in Table A suggest that in lakes with pH < 8 ΔC_{CO2} is an excellent estimator of ΔC_{DIC}, with ΔC_{CO2} underestimating ΔC_{DIC} by less than 10%. However, in lakes with pH = 8 ΔC_{CO2} is already ~ 20% smaller than ΔC_{DIC}, i.e. about 20% of the change in DIC is channeled to HCO_3^- and HCO_3^{--}. With increasing pH the difference between ΔC_{CO2} and ΔC_{DIC} increases rapidly, e.g. at pH = 8.5 ΔC_{CO2} is ~5 times smaller than ΔC_{DIC}. Hence, in lakes with pH <8 ΔC_{DIC} can be estimated as ΔC_{CO2} but in lakes with pH ≥ 8 the assessment of ΔC_{DIC} requires consideration of the carbonate balance.

Estimates of ΔC_{DIC} utilizing C_{CO2} and pH measurements

The carbonate balance can be considered in the estimation of ΔC_{DIC} by utilizing pH data in addition to C_{CO2} measurements. However, this technique only improves the estimate of ΔC_{DIC}, $\Delta C_{DIC,e\_pH}$, if the uncertainty of the pH data is very low. We tested the sensitivity of $\Delta C_{DIC,e\_pH}$ to errors in pH assuming that the measured pH agrees with the true pH at low C_{DIC} but exceeds the true pH value at high C_{DIC} by 0.005. DIC was assumed to increase due to respiration by ΔC_{DIC} = 0.01 mmol L^{-1}, ΔC_{DIC} = 0.02 mmol L^{-1}, and ΔC_{DIC} = 0.04 mmol L^{-1}, respectively. The difference between $\Delta C_{DIC,e\_pH}$ and the correct change in DIC is small for waters with pH <8 (Table B). However, at pH ≥ 8 the very small error in pH of 0.005 leads to estimates of the change in DIC $\Delta C_{DIC,e\_pH}$ that exceed the true ΔC_{DIC} by up to several hundred percent and the ratio $\Delta C_{DIC,e\_pH}$ / ΔC_{DIC} increases the smaller the true change in DIC. In contrast to the uncertainty in pH measurements, the absolute precision of the measured pH has essentially no effect on the estimated $\Delta C_{DIC,e\_pH}$. If pH is shifted by 0.005 at low and high values of C_{DIC} the ratio $\Delta C_{DIC,e\_pH}$ / ΔC_{DIC} is ~100% in all cases.

Table B. Sensitivity of estimates of $\Delta C_{DIC,ph}$ to uncertainties in pH measurements.
The table provides the ratio $\Delta C_{DIC,e\_pH}$ / ΔC_{DIC} in %. $\Delta C_{DIC,ph}$ were calculated assuming that the pH data are correct at low C_{DIC} but deviate by 0.005 from the correct pH at high C_{DIC}.

metabolism ΔC_{DIC} mmol L^{-1}	pH				
	6.0	7.0	8.0	8.3	8.5
0.01	101	109	192	288	404
0.02	101	105	146	194	252
0.04	100	102	123	147	176

Estimates of ΔC_{DIC} utilizing C_{CO2} and alkalinity measurements

In the diel CO_2-technique estimates of ΔC_{DIC} are not based on measurements of C_{CO2} and pH but on measured C_{CO2} and carbonate alkalinity which is assumed to remain constant. In the following we test the sensitivity of the estimates of ΔC_{DIC} to changes in alkalinity. The calculations involve two steps: First, the true C_{CO2} is calculated for the initial condition and the condition after the change in C_{DIC} and alkalinity in the respective scenario. Then, these C_{CO2} concentrations are considered as the measured C_{CO2} and the change in C_{DIC} , $\Delta C_{DIC,e\_ALK}$ is estimated from these values assuming that the initial alkalinity applies to the conditions before and after the changes assumed in the scenario. As above, the scenarios assume ΔC_{DIC} = 0.01 mmol L^{-1}, ΔC_{DIC} = 0.02 mmol L^{-1}, and ΔC_{DIC} = 0.04 mmol L^{-1}, respectively. Alkalinity was assumed to increase in parallel with the increase in C_{DIC}. In the sensitivity study we considered changes in alkalinity of ΔALK_{Carb} = 0.005 $mmol_{eq}$ L^{-1}, ΔALK_{Carb} = 0.01 $mmol_{eq}$ L^{-1}, and ΔALK_{Carb} = -0.005 $mmol_{eq}$ L^{-1}, respectively. ΔALK_{Carb} = 0.005 $mmol_{eq}$ L^{-1} corresponds to an uptake of 5 µmol L^{-1} NO_3^- i.e. ~10% of the total NO_3^- concentration in Lake Illmensee.

Two different causes for alkalinity changes were considered: a) a change in H_3O^+ -ions, e.g. connected to the uptake or release of non-carbonate ions$^-$, and b) a change in carbonate ions due to calcite precipitation or dissolution of solid carbonates. In the latter case, not only alkalinity changes but also C_{DIC}.

a) Alkalinity change due to uptake or release of non-carbonate ions

An increase in carbonate alkalinity due to uptake or release of non-carbonate ions implies a decrease in H_3O^+ ions, i.e. a shift towards higher pH. In this case the concentration of CO_2 is

reduced in favor of the concentration of bicarbonate and carbonate ions. Hence, an increase in C_{DIC} due to respiration and a simultaneous increase in alkalinity due to uptake or release of non-carbonate ions results in a smaller change in CO_2 as would be expected under constant alkalinity. The diel CO_2-technique based on measured CO_2 concentrations and the assumption of constant alkalinity therefore underestimates the true increase in C_{DIC} due to respiration if alkalinity increases with increasing C_{DIC} (Table C).

Table C. Sensitivity of estimated ΔC_{DIC} to changes in alkalinity due to uptake or release of non-carbonate ions. Scenarios considered assume that metabolic processes cause a change in DIC and that other processes cause an additional change in ALK_{Carb}. The table provides the ratio of ΔC_{DIC} estimated with the diel CO_2 technique to the true ΔC_{DIC} applied in the scenario: $\Delta C_{DIC\_e\_ALK} / \Delta C_{DIC}$. Values are in %.

metabolism ΔC_{DIC} mmol L^{-1}	ΔALK_{Carb} mmol$_{eq}$ L^{-1}	pH				
		6.0	7.0	8.0	8.3	8.5
0.01	0.005	55	50	51	52	53
0.02	0.005	78	75	75	76	76
0.04	0.005	90	88	88	88	88
0.02	0.01	54	50	51	51	52
0.02	-0.005	118	125	125	124	124

If alkalinity does not change with C_{DIC} but is systematically over- or underestimated, the calculated ΔC_{DIC} agrees very well with the true ΔC_{DIC} (i.e. within 2% for all scenarios).

b) Alkalinity change due to dissolution of solid carbonates and calcite precipitation

The dissolution of solid carbonate also leads to an increase in alkalinity. In this case not only alkalinity but also C_{DIC} increases, whereby the molar change in C_{DIC} is half of the molar change in carbonate alkalinity. However, the reduction of the CO_2 concentration due to the decrease in acidity outweighs the increase in CO_2 due to the release of DIC from solid carbonates. Overall, an increase in alkalinity and C_{DIC} due to the dissolution of solid carbonates and a simultaneous increase in DIC due to respiration results in a change in C_{CO2} that is smaller than would be expected for a constant alkalinity but larger than in the case above, in which alkalinity changes were associated with changes in the concentration of HO_3^+ but not in DIC. Hence, the diel CO_2-technique based on measured CO_2 concentrations and the assumption of

constant alkalinity underestimates the true change in DIC associated with respiration if alkalinity increases with increasing DIC (Table D).

Table D. Sensitivity of estimated ΔC_{DIC} to changes in alkalinity due to dissolution of solid carbonates. Scenarios assume that metabolic processes cause a change in DIC and that calcite precipitation or dissolution of solid carbonates cause an additional change in ALK_{Carb} and in DIC. The molar change in DIC due to the transformations of carbonates must be half of that in ALK_{Carb}. The table provides the ratio of ΔC_{DIC} estimated with the diel CO_2-technique to the true ΔC_{DIC} applied in the scenario: $\Delta C_{DIC\_estim}/\Delta C_{DIC}$. Values are in %.

metabolism ΔC_{DIC} mmol L^{-1}	ΔALK_{Carb} $mmol_{eq}$ L^{-1}	transformation of solid carbonates ΔC_{DIC} mmol L^{-1}	pH				
			6.0	7.0	8.0	8.3	8.5
0.01	0.005	0.0025	80	75	76	77	78
0.02	0.005	0.0025	91	88	88	88	89
0.04	0.005	0.0025	96	94	94	94	94
0.02	0.01	0.005	80	75	76	76	77
0.02	-0.005	-0.0025	105	112	112	112	111

In case of calcite precipitation alkalinity decreases due to a reduction in carbonate ions. This implies more acidic conditions and thus causes a shift to higher CO_2 concentrations at the expense of bicarbonate and carbonate. In addition to the change in alkalinity, calcite precipitation reduces DIC whereby the molar change in C_{DIC} is half of the molar change in alkalinity. In case of calcite precipitation the decrease in CO_2 resulting from a loss in DIC due to production is smaller than in the case of constant alkalinity. Hence, the diel CO_2-technique underestimates production if production is accompanied by calcite precipitation.

Summarizing, in case that alkalinity changes, the diel CO_2-technique assuming constant alkalinity underestimates metabolic rates if the changes in DIC due to metabolic processes have the same sign as changes in alkalinity, and overestimates metabolic rates if the changes in DIC due to metabolic processes and alkalinity have opposite signs. Alkalinity changes caused by calcite precipitation or carbonate dissolution have a smaller effect on the estimates of metabolic rates than the same alkalinity change caused by other ions. However, as in manly lakes alkalinity

is dominated by carbonate ions, calcite precipitation may be the primary cause of substantial changes in alkalinity.
