# Supplementary material for: Lake Metabolism: Comparison of Lake Metabolic Rates Estimated from a Diel CO2- and the Common Diel O2-Technique
Source: PLoS One. 2016 Dec 21;11(12):e0168393. doi: 10.1371/journal.pone.0168393 (PMC5176309; doi:10.1371/journal.pone.0168393)
Supplement: S8 Appendix — (PDF) [file pone.0168393.s008.pdf]

S8 Appendix: Compilation of the main equations of the CO_2- and the O_2-technique

Table. Comparison of the main equations of the CO_2- and the O_2-technique.

Open water O_2-technique	Open water CO_2-technique
**One dimensional transport –reaction equation considering metabolism and vertical transport and assuming horizontally homogeneous conditions:**	
$\frac{\partial C_{O2}}{\partial t} = GPP_O - R_O - \frac{1}{A}\frac{\partial(A \cdot F_{O2})}{\partial z} + \frac{1}{A}\frac{\partial A}{\partial z} F_{O2,sed}$ $Boundary\ conditions:$ $F_{O2,surf} = v_{O2} \cdot (C_{O2} - C_{O2,equ})$ $F_{O2,bot} = F_{O2,sed}$	$\frac{\partial C_{DIC}}{\partial t} = -GPP_C + R_C - \frac{1}{A}\frac{\partial(A \cdot F_{DIC})}{\partial z} + \frac{1}{A}\frac{\partial A}{\partial z} F_{DIC,sed}$ $Boundary\ conditions:$ $F_{DIC,surf} = F_{CO2,surf} = v_{CO2} \cdot (C_{CO2} - C_{CO2,equ})$ $F_{DIC,bot} = F_{DIC,sed}$
**Definition of the lake respiration rate:**	
$R_{L\_O} = R_O - \frac{1}{A}\frac{\partial A}{\partial z} F_{O2,sed}$	$R_{L,C} = R_C + \frac{1}{A}\frac{\partial A}{\partial z} F_{DIC,sed}$
**Lake net production:**	
$NEP_{L\_O} = \frac{\partial C_{O2}}{\partial t} + \frac{1}{A}\frac{\partial(A \cdot F_{O2})}{\partial z}$	$NEP_{L\_C} = -\frac{\partial C_{DIC}}{\partial t} - \frac{1}{A}\frac{\partial(A \cdot F_{DIC})}{\partial z}$
**Lake gross production:**	
$GPP_{L\_O} = NEP_{L\_O} + R_{L\_O}$	$GPP_{L\_C} = NEP_{L\_C} + R_{L\_C}$

Table. Continued

**Open water O_2-technique**	**Open water CO_2-technique**

Estimation of lake net production for different assumptions on the fluxes:

(i) $NEP_{L\_O} = +\frac{\partial C_{O2}}{\partial t}$

$NEP_{L\_C} = -\frac{\partial C_{DIC}}{\partial t}$

(ii) $NEP_{L\_O,A} = +\frac{\partial C_{O2}}{\partial t} + \frac{F_{O2,atm} \cdot A_0}{V_{mix}}$

$NEP_{L\_C,A} = -\frac{\partial C_{DIC}}{\partial t} - \frac{F_{CO2,atm} \cdot A_0}{V_{mix}}$

(iii) $NEP_{L\_O,F} = +\frac{\partial C_{O2}}{\partial t} + \frac{A_0 \cdot F_{O2,atm} - A_{Zmix} \cdot F_{O2,Zmix}}{V_{Zmix}}$

$NEP_{L\_C,F} = -\frac{\partial C_{CO2}}{\partial t} - \frac{A_0 \cdot F_{CO2,atm} - A_{Zmix} \cdot F_{CO2,Zmix}}{V_{Zmix}}$

(iv) $NEP_{L\_O,D} = +\frac{\partial C_{O2}}{\partial t} + \frac{A_0 \cdot F_{O2,atm} - A_{Zmix} \cdot F_{O2,turb}}{V_{Zmix}}$

$NEP_{L\_C,D} = -\frac{\partial C_{CO2}}{\partial t} - \frac{A_0 \cdot F_{CO2,atm} - A_{Zmix} \cdot F_{CO2,turb}}{V_{Zmix}}$

Estimation of the lake respiration rate:

$R_{L\_O,night} = -\frac{1}{\Delta t_{night}} \int_{t_{s,night}}^{t_{e,night}} NEP_{L\_O}(t') \cdot dt'$

or

$C_{O2,\mathrm{mod}}(t) = C_{O2}(t) + \int_{t_{s,night}}^{t} \frac{1}{A} \frac{\partial \left(A \cdot F_{O2}(t') \right)}{\partial z} \cdot dt'$

$C_{O2,\mathrm{mod}}(t) = a_{L\_O} - R_{L\_O,nightfit} \cdot t \quad and \quad t_{s,night}$

$R_{L\_O} = R_{L\_O,night}$

$R_{L\_C,night} = -\frac{1}{\Delta t_{night}} \int_{t_{s,night}}^{t_{e,night}} NEP_{L\_C}(t') \cdot dt'$

or

$C_{DIC,\mathrm{mod}}(t) = C_{DIC}(t) + \int_{t_{s,night}}^{t} \frac{1}{A} \frac{\partial \left(A \cdot F_{DIC}(t') \right)}{\partial z} \cdot dt'$

$C_{DIC,\mathrm{mod}}(t) = a_{L\_C} + R_{L\_C,nightfit} \cdot t \quad and \quad t_{s,night} <$

$R_{L\_C} = R_{L\_C,night}$

Estimation of lake gross production:

$GPP_{L\_O} = NEP_{L\_O} + R_{L\_O}$

$GPP_{L\_C} = NEP_{L\_C} + R_{L\_C}$
