# Supplementary material for: Lake Metabolism: Comparison of Lake Metabolic Rates Estimated from a Diel CO2- and the Common Diel O2-Technique
Source: PLoS One. 2016 Dec 21;11(12):e0168393. doi: 10.1371/journal.pone.0168393 (PMC5176309; doi:10.1371/journal.pone.0168393)
Supplement: S9 Appendix — (PDF) [file pone.0168393.s009.pdf]

S9: Compilation of the empirical relations used in this study

Table. Empirical relations used in this study.

Equilibrium between the different carbon species

Carbon alkalinity	$ALK_{Carb}=C_{HCO3-}+2\cdot C_{CO3--}+C_{OH-}-C_{H3O+}$<br>$C_{DIC}=C_{CO2}/\alpha_0(pH,T,S)$; $C_{HCO3-}=C_{DIC}\cdot\alpha_1(pH,T,S)$; $C_{CO3--}=C_{DIC}\cdot\alpha_2(pH,T,S)$<br>$ALK_{Carb}=C_{CO2}/\alpha_0(pH,T,S)\cdot(\alpha_1(pH,T,S)+2\cdot\alpha_2(pH,T,S))+(K_W\cdot 10^{pH}-10^{-pH})\cdot 1000$	[1]
α_0	$\alpha_0=\left(1+\frac{K_1}{H^+}+\frac{K_1\cdot K_2}{(H^+)^2}\right)^{-1}$	[1]
α_1	$\alpha_1=\left(\frac{H^+}{K_1}+1+\frac{K_2}{H^+}\right)^{-1}$	[1]
α_2	$\alpha_2=\left(\frac{(H^+)^2}{K_1\cdot K_2}+\frac{H^+}{K_2}+1\right)^{-1}$	[1]
K_1	$\ln(K_1)=290.9097-\frac{14554.21}{T}-45.0575\cdot\ln(T)$<br>$+\left(-228.39774+\frac{9714.36839}{T}+34.485796\cdot\ln(T)\right)\cdot S^{0.5}+\left(54.20871-\frac{2310.48919}{T}-8.19515\cdot\ln(T)\right)\cdot S^1$<br>$+\left(-3.969101+\frac{170.22169}{T}+0.603627\cdot\ln(T)\right)\cdot S^{1.5}-0.00258768\cdot S^2$	[2]
K_2	$\ln(K_2)=207.6548-\frac{11843.79}{T}-33.6485\cdot\ln(T)$<br>$+\left(-167.69908+\frac{6551.35253}{T}+25.928788\cdot\ln(T)\right)\cdot S^{0.5}+\left(39.75854-\frac{1566.13883}{T}-6.171951\cdot\ln(T)\right)\cdot S^1$<br>$+\left(-2.892532+\frac{116.270079}{T}+0.45788501\cdot\ln(T)\right)\cdot S^{1.5}-0.00613142\cdot S^2$	[2]

Dissociation of water

Dissociation	$H^+ = 10^{(-pH)}$ [mol L^{-1}] $OH^- = K_w./H^+$ [mol L^{-1}]	
K_W	$\ln(K_w) = 148.9802 - \frac{13847.26}{T} - 23.6521 \cdot \ln(T) + \left(-5.977 + \frac{118.67}{T} + 1.0495 \cdot \ln(T)\right) \cdot S^{0.5} - 0.01615 \cdot S$ and T [K]; S [g kg^{-1}]	[2]

Gas exchange with the atmosphere

Gas flux to the atmosphere	$F_{surf} = v_{gas} \cdot (C - C_{equ})$	
Concentration of CO_2 in atmospheric equilibrium	$C_{CO2,equ} = p_{CO2} \cdot Henry_{CO2}$ $Henry_{CO2}$ = exp {-58.0931 + 90.5069 · (100 / T) + 22.294 · ln(T/100) ... + S · (0.027766 + (-0.025888 · (T/100) + 0.0050578 · (T/100)^2)} [mol L^{-1} atm^{-1}] T in [K] and S in [g kg^{-1}] p_{CO2} = 400 ppm · 0.924 atm is the atmospheric partial pressure of CO_2 at Lake Illmensee	[3]
Concentration of O_2 in atmospheric equilibrium	$C_{O2,equ}$ = exp { -177.7888+255.5907· (100./T) + 146.4813 · ln(T/100) - 22.204 · (T/100) + S · (-0.037362 + 0.016504 · (T/100) - 0.0020564 · (T/100)^2)} · (p-p_s)/(1-p_s)/1000 [ccSTP g^{-1}] p = 0.924 atm is the local atmospheric pressure at Lake Illmensee in [atm]. $p_s = 10^{(0.7859+0.03477*Tc)./(1+0.00412*Tc)}$ / 1013.25 [atm]; is the saturation water vapor pressure in [atm] at the water temperature Tc in [°C] $C_{O2,equ}$ [mol L^{-1}] = $C_{O2,equ}$ [ccSTP g^{-1}] · 4.4615 10^{-2} [mol L^{-1} / ([ccSTP g^{-1})]	[4]
Gas exchange velocity	$v_{g,Sc} = v_{g,600} \cdot \left(\frac{Sc}{600}\right)^a$ $a = \begin{cases} 2/3 \text{ if } U_{10} \le 3.7 ms^{-1} \\ 1/2 \text{ if } U_{10} > 3.7 ms^{-1} \end{cases}$ $v_{g,600} = 2.07 + 0.215 \cdot U_{10}^{1.7}$ cm/h and U_{10} is in [m s^{-1}] [6] Sc represents the Schmidt number of the gas. Sc were determined from the empirical relations of [7].	[5]

Vertical mixing of dissolved oxygen

Turbulent diffusive flux	$F_{O2,turb} = -K_z \frac{dC_{O2}}{dz}$
Turbulent diffusivity	$K_z = 8.167 \cdot 10^{-8} \cdot A_S^{0.56} \cdot \left(N^2\right)^{-0.43}$ [m^2 s^{-1}] [8]<br>The lake surface area A_S is in [km]. The Brunt-Väisälä frequency N is in [s^{-1}] and calculated from<br>$N^2 = -g \cdot \frac{1}{\rho} \frac{d\rho}{dz}$<br>The vertical coordinate z increases in the upward direction.<br>ρ is the density of water calculated from [9].
Mixed layer deepening	$F_{O2,deepen} = \frac{1}{\Delta t} \frac{1}{A_{Zmix}} \left(\frac{1}{V_{Zmix(2)}} \int_{-Zmix(2)}^{0} A \cdot C_{O2} \cdot dz' - \frac{1}{V_{Zmix(1)}} \int_{-Zmix(1)}^{0} A \cdot C_{O2} \cdot dz' \right)$<br>$A_{Zmix}$, is the area of the cross section at Z_{mix} and V_{Zmix} is the volume of the mixed surface layer. The oxygen profile at time 1, C_{O2}, is integrated to Z_{mix} at time 1, Z_{mix} (1), and to Z_{mix} after the time interval Δt, i.e. to Z_{mix}(2) at time 2. The time interval Δt was chosen to be 1 hour.

Lake properties

Surface Area	$A_S = 6.52574 \cdot 10^5$ m^2
Volume of the entire lake	$V = 5.1655 \cdot 10^6$ m^3
Maximum depth	$d_{max} = 15.7$ m
Local air pressure:	$p = 93600$ Pa

References:

1. Stumm W, Morgan JJ. Aquatic Chemistry, Chemical Equilibria and Rates in Natural Waters. 3rd. ed. John Wiley & Sons, Inc., New York; 1996. 1022p p.

2. Millero FJ. Thermodynamics of the carbon dioxide system in the oceans. Geochim Cosmochim Acta. 1995;59(4):661–77.

3. Weiss RF. Carbon dioxide in water and seawater: the solubility of a non-ideal gas. Mar Chem. 1974;2:203–15.

4. Weiss RF. The solubility of nitrogen, oxygen and argon in water and seawater. Deep Res. 1970;17:721–35.

5. Liss PS, Merlivat L. Air−sea gas exchange rates: Introduction and synthesis. In: Reidel D, editor. The Role of Air−Sea Exchange in Geochemical Cycling. Publishing Company, Dordrecht, The Netherlands; 1986. p. 113–27.

6. Cole JJ, Caraco NF. Atmospheric exchange of carbon dioxide in a low-wind oligotrophic lake measured by the addition of SF6. Limnol Oceanogr. 1998;43(4):647–56.

7. Wanninkhov K. Relationship between wind speed and gas exchange. J Geophys Res Ocean. 1992;97:7373–82.

8. Hondzo M, Stefan HG. Lake water temperature simulation model. J Hydraul Eng. 1993;119:1251–73.

9. Chen C-TA, Millero FJ. Precise thermodynamic properties for natural waters covering only the limnological range. Limnol Oceanogr. 1986;31(3):657–62.
